# Pulmonary diffusing capacity and dyspnoea following COVID-19: Insights from multicentre datasets

**DOI:** 10.1016/j.dib.2025.111925

**Published:** 2025-07-25

**Authors:** Gerald S. Zavorsky, Giovanni Barisione, Thomas Gille, Roberto W. Dal-Negro, Marta Núñez-Fernández, Leigh Seccombe, Gianluca Imeri, Fabiano Di Marco, Jann Mortensen, Elisabetta Salvioni, Piergiuseppe Agostoni, Vito Brusasco

**Affiliations:** a Department of Physiology and Membrane Biology, University of California Davis, Sacramento, CA, United States; b Struttura Semplice Fisiopatologia Respiratoria, Clinica Malattie Respiratorie e Allergologia, IRCCS Ospedale Policlinico San Martino, Genoa, Italy; c Department of Physiology and Functional Exploration, University Hospitals of Paris Seine-Saint-Denis, HP–AP, Bobigny, France; d CESFAR – National Center for Respiratory Pharmacoeconomics and Pharmacoepidemiology, 37124, Verona, Italy; e Service of Pneumology, University Hospital Complex of Vigo, Vigo, Spain; f NeumoVigo, Institute of Health Research South Galicia (IISGS) 36213, Vigo, Spain; g The University of Sydney, Camperdown, NSW, Australia; h Thoracic Medicine, Concord Repatriation General Hospital, Concord, NSW, Australia; i Department of Health Sciences, University of Milano, ASST Papa Giovanni XXIII Hospital, Bergamo, Italy; j Department of Clinical Physiology and Nuclear Medicine, Rigshospitalet, Copenhagen University Hospital, Copenhagen, Denmark; k Centro Cardiologico Monzino IRCCS, Milan, Italy; l Department of Clinical Sciences and Community Health, Cardiovascular Section, University of Milan, Milan, Italy; m Department of Experimental Medicine, University of Genoa, Genoa, Italy

**Keywords:** COVID-19, SARS CoV-2, COVID-19, Pulmonary function, Diffusing capacity, NO-CO double diffusion, Lung diagnostics

## Abstract

Pulmonary complications remain a significant challenge for COVID-19 survivors, necessitating advanced diagnostic approaches for long-term assessment. We present a curated, open-access dataset of pulmonary function measurements—including nitric oxide (DLNO) and carbon monoxide (DLCO) diffusing capacities—in 572 post–COVID-19 patients and 72 healthy controls (filtered from an original cohort of 726 survivors and 126 controls). Collected across eight international centres, the data include demographics, spirometry, lung volumes, and 5–6 s single-breath DLNO_5s_, DLCO_5s_, and alveolar volume (VA_5s_). Missing values for total lung capacity were imputed, and low-quality or system-specific (Hyp’Air Compact) measurements were excluded in the filtered dataset. A third subset (333 patients, 54 controls) links these measurements to dyspnoea severity (mMRC scale) for correlation and proportional odds analyses. This resource underpins predictive modeling of post–COVID pulmonary impairment via summed z-scores (DLNO + DLCO) and aims to accelerate validation of NO-CO diagnostics. The freely accessible datasets are provided in both SPSS (.sav) and .csv formats at the Mendeley Data Cloud-based repository and includes nominal, ordinal, and scalar data.

Specifications Table

**Table:** 

Subject	Health *and* Medical Sciences
Specific subject area	Pulmonary and Respiratory Medicine*.*
Type of data	Filtered data in SPSS (.sav) and .csv formats
Data collection	Lung function measured using NO—CO diffusion technique, standardized with reference equations from Zavorsky & Cao [20] and Munkholm et al. [24]. Spirometry and total lung capacity were standardized using reference equations for Whites from the Global Lung Function Initiative (GLI)
Data source location	Hospitals in Genoa, Verona, Milan, Bergamo (Italy), Vigo (Spain), Bobigny (France), Copenhagen (Denmark), Sydney (AUS)
Data accessibility	Repository name: Mendeley DataData identification number: 10.17632/92gvt9vmrm.3 Direct URL to data: https://data.mendeley.com/datasets/92gvt9vmrm/3This is the 3rd version of the data. The clarity of the data is enhanced with more appropriate labelling. The data are in two formats: A) Three files in SPSS (.sav) format. Once the file is open you will find the labels for each parameter under the “VARIABLE VIEW” tab. B) Three files in csv format.
Related research article	Zavorsky G.S., Barisione G., Gille T., Dal-Negro R.W., Núñez-Fernández M., Seccombe L., Imeri G., Di Marco F., Mortensen J., Salvioni E., Agostoni P., Brusasco V. (2025). Enhanced detection of patients with previous COVID-19: Superiority of the double diffusion technique. BMJ Open Respir Res https://dx.doi.org/10.1136/bmjresp-2024-002561

## 1 Value of the Data

• A most extensive international collection of simultaneous DLNO and DLCO measurements in post–COVID cohorts.
• Facilitates reproducibility and cross-center validation of diagnostic models using combined z-scores.
• Enables research into membrane vs. red blood cell diffusion deficits after SARS-CoV-2 infection.
• Supports linking objective diffusion impairments to dyspnoea severity (mMRC) for clinical correlation.
• Provides benchmark data for personalized medicine and longitudinal research on respiratory diseases.
• Enhances meta-analyses and systematic reviews on long-term pulmonary outcomes of COVID-19 and other respiratory illnesses.

## 2 Background

Since the initial outbreak of severe acute respiratory syndrome coronavirus 2 (SARS-CoV-2) in late 2019, coronavirus disease 2019 (COVID-19) has emerged as not only an acute respiratory illness but also a condition with significant long-term pulmonary sequelae. A substantial proportion of survivors report persistent dyspnoea, exercise intolerance, and reduced quality of life months after viral clearance. Objective measurements corroborate these symptoms, revealing nearly 50 % of patients have reduced pulmonary function, such as decreased forced vital capacity (FVC), restrictive spirometry pattern, and impaired diffusing capacity for carbon monoxide (DLCO) at four months post-infection [1]. A meta-analysis demonstrated that up to 60 % of patients with previous COVID-19 have DLCO values below 80 % predicted [2]. However, standard DLCO alone cannot fully disentangle the relative contributions of alveolar membrane conductance and pulmonary capillary blood volume to gas transfer abnormalities.

The NO—CO double diffusion technique offers a solution by simultaneously measuring pulmonary diffusing capacity for nitric oxide (DLNO) and DLCO during a single breath-hold. Nitric oxide binds rapidly to hemoglobin and is primarily limited by membrane conductance, whereas carbon monoxide diffusion is influenced by both membrane and red blood cell factors [3]. By applying established reference equations, separate estimates of membrane diffusing capacity (DM) and capillary blood volume (Vc) can be derived, yielding a more granular picture of gas exchange dysfunction. When several cardiopulmonary diseases are grouped, sensitivity can be higher by 12 % [95 % CI = 1 to 23 %] when DLNO z-scores were used to identify impairment compared DLCO z-scores when compared to matched controls (*p* = 0.0474) [4]. Recently, combining DLNO+DLCO z-scores resulted in better prediction and classification of patients with heart failure compared to matched controls [5].

Despite its potential, the adoption of the NO—CO technique in routine clinical practice has been limited by a lack of standardized protocols, the sparse availability of dual-gas analysers, and regulatory barriers in different regions. Clinicians remain largely unfamiliar with DLNO metrics, and the integration of reference values into commercial pulmonary function software is inconsistent.

To address these gaps and accelerate translational uptake, we have assembled a large, multicentre dataset that integrates raw and quality-controlled DLNO and DLCO measurements from COVID-19 survivors and matched healthy controls. Our pooled cohort of 572 patients and 72 controls (filtered from an unfiltered pool of 726 survivors and 126 controls) captures diverse demographics and disease severities across six international centres. By providing both raw values (DLNO_5s_, DLCO_5s_, VA_5s_) and fitted lung function z-scores, quality control criteria, and imputation methods, this dataset empowers researchers to validate membrane- and capillary-specific diagnostic models [6]. Furthermore, a dyspnoea-linked subset (333 patients, 54 controls) enables a direct correlation of physiological impairments with patient-reported outcomes (MRC scale), supporting the development of clinically actionable thresholds. The results of these analyses are found in our companion research article examining prediction and classification using the NO—CO double diffusion technique in patients with previous COVID-19 [7].

In summary, this resource builds upon foundational NO‑CO diffusion research to meet an urgent need for comprehensive, open-access data in post-COVID pulmonary assessment. It lays the groundwork for personalized diagnostics, targeted interventions, and a deeper understanding of the long-term mechanisms of lung injury following SARS-CoV-2 infection.

## 3 Experimental Design, Materials, and Methods

This companion paper provides results of an Individual participant data (IPD) meta-analysis published in BMJ Open Respiratory Research [7]. An IPD meta-analysis is a method of synthesizing data from multiple studies by combining the raw, participant-level data instead of relying solely on summary statistics. This approach involves obtaining the original datasets from each study and re-analysing them together in a consistent framework. It is considered one of the most rigorous methods of data synthesis because it enables harmonization of variables and analytical methods across studies.

After contacting authors via email, we were able to obtain 841 cases from eight hospital centres: 48 cases from Verona, IT [8]; 165 cases from Genova IT [9,10], 190 cases from Vigo, ESP [11]; 66 cases from Bobigny, FR [12,13], including 10 unpublished cases; 21 cases from Sydney, AUS [14] 32 cases from Bergamo, IT [15], 129 cases from Copenhagen, DK [16], 190 cases from Milan, IT [17] including controls [18].

To ensure measurement comparability, we excluded all data collected using the Hyp’Air Compact device [8,14], which systematically raises DLNO and alveolar volume compared to the MasterScreen PFT Pro system [19,20]. This exclusion removed 69 subjects.

We subsequently removed cases that had measurements that were not physiological:

• FVC ≥ TLC
• VA_5s_ ≥ TLC by ≥ 0.20 L
• FEV₁/FVC > 0.95
• Control group fitted z-scores outside ± 2.50
• Non-white participants (excluded due to the lack of validated DLNO reference equations in non-white populations)

After these exclusions, the final pooled dataset comprised nine published studies and 10 control subjects from Dr. Gille’s dataset, resulting in a total of 572 post-COVID participants and 72 controls, never infected with SARS-CoV-2 (**Covid19_&_Controls_Filtered.sav**) [6].

Participants underwent a comprehensive battery of lung function tests. The NO—CO double diffusion technique was used to measure diffusing capacities for nitric oxide (DLNO_5s_) and carbon monoxide (DLCO_5s_ during a single-breath manoeuvre lasting 5–6 s. Alveolar volume (VA_5s_) was simultaneously measured. Spirometry was performed to measure forced expiratory volume in one second (FEV_1_), forced vital capacity (FVC), and the FEV_1_/FVC ratio to detect obstructive patterns. Whole body plethysmography was used to measure total lung capacity (TLC), identifying restrictive lung impairments.

Lung function variables were standardized into z-scores using reference equations to account for age, sex, height, and testing equipment. The Global Lung Function Initiative (GLI) reference equations were applied for spirometry, DLCO, and TLC [[21], [22], [23]], while The NO—CO double diffusion technique obtained DLNO, DLNO, and VA z-scores were derived from previously established reference equations specific to the Jaeger MasterScreen Pro pulmonary function systems [20,24].

### 3.1 Imputation of missing values for total lung capacity (TLC)

To address approximately 190 missing Total Lung Capacity (TLC) values in our pooled dataset of 841 subjects, we employed multiple imputation using chained equations (MICE), a well-established and flexible method for handling incomplete multivariate data under the assumption that data were missing at random (MAR). Imputation was conducted using the mice package (version 3.16.0) in R (version 4.3.2), generating five imputed datasets via predictive mean matching (PMM). This approach preserves the distributional characteristics of the observed data and avoids implausible imputed values by drawing from cases with similar covariate profiles. The imputation model included six predictors: age, height, weight, FVC, disease status (coded 0 for never-infected and 1 for post-COVID), and study site. These variables were selected based on their known clinical associations with lung volume and their predictive capacity for TLC. Sex was initially considered but excluded from the final model due to a lack of significance in preliminary testing.

To assess the coherence between imputed values and physiologic expectations, we developed a linear regression model using only complete cases with observed TLC data. The model regressed TLC on the same set of predictors: age, height, weight, FVC, disease status, and study site. Predicted TLC values from this model were compared to the mean of the five imputed TLC values for everyone.

Following imputation, the five TLC estimates for each subject were averaged to generate a single composite value (Mean TLC) for use in all subsequent analyses and visualizations. We performed multiple evaluations to verify the quality of the imputation. Additionally, residuals were examined across disease status and study site.

### 3.2 Impact of DLNO/DLCO ratio and time since SARS-CoV-2 on lung function z-scores

We evaluated the impact of prior COVID-19 on the DLNO/DLCO ratio using a generalized linear mixed model (GLMM) with a Gamma distribution and log link, implemented via the *glmer* function in the lme4 R package. The DLNO/DLCO ratio served as the outcome variable, with COVID-19 history included as a fixed effect and the study site modelled as a random intercept to account for between-site variability. Model parameters were estimated using maximum likelihood via the Laplace approximation and reported as log ratios, which were subsequently back-transformed to yield multiplicative effects with corresponding 95 % confidence intervals.

To explore the association between time since SARS-CoV-2 infection and pulmonary function, we analysed data from participants with a history of COVID-19 (median time from diagnosis to testing: 130 days; range: 32–575 days). Separate linear mixed-effects models were fitted for each of the 18 lung-function z-scores using the *lmer* function from the lmerTest package. In these models, days since diagnosis were specified as a fixed effect, with the study site again treated as a random intercept. For each outcome, we report the fixed-effect estimate, standard error, Satterthwaite’s degrees of freedom, and p-value, quantifying the average per-day change in pulmonary function metrics following SARS-CoV-2 infection.

### 3.3 Model selection and classification metrics

Statistical analysis was conducted using both frequentist [23,25] and Bayesian [26] frameworks. Mixed-effects logistic regression models were employed to identify predictors of post-COVID-19 pulmonary impairments, with study centres treated as random intercepts to account for variability across sites. Frequentist analysis used the Bayesian Information Criterion (BIC) for model selection. Bayesian analysis was performed using Markov Chain Monte Carlo (MCMC) methods to generate posterior distributions for model parameters, with the Leave-One-Out Information Criterion (LOOIC) employed for model comparison. Model performance was evaluated using the area under the receiver operating characteristic curve (AUROC) and Matthews Correlation Coefficient (MCC) [[27], [28], [29]]. The 95 % Confidence Interval (CI) for the Matthews Correlation Coefficient (MCC) was calculated and assessed based on repeated k-fold cross-validation, using a threshold of 0.5 with 10 folds and 10 repetitions [30]. Z-scores from the NO—CO double diffusion technique were also summed “combined” for one overall z-score (DLNO_5s_ z-score + DLCO_5s_ z-scores = summed z-score).

### 3.4 Agreement analyses

Cohen’s kappa was calculated to assess the agreement between classifications of DLCO₅_s_ and DLNO₅_s_ values falling below the lower limit of normal (LLN). To evaluate the reliability of the agreement, 95 % confidence intervals were derived using bootstrap resampling. Interpretation of kappa values followed established benchmarks for assessing the strength of agreement [31].

### 3.5 Logistic regression and PCA analyses

This analysis aimed to examine the relationship between pulmonary diffusion metrics and prior COVID-19 infection using logistic regression models and to explore the utility of Principal Component Analysis (PCA) for dimensionality reduction and multicollinearity mitigation. The models were built using z-scores for three key lung diffusion variables—DLNO_5s_, DLCO_5s_, and VA_5s_ —standardized using two methods: segmented linear regression (SLR) and generalized additive models for location, scale, and shape (GAMLSS), both based on the reference equations by Zavorsky & Cao (2022) [20]. The analytic dataset comprised 644 complete cases across several international study sites, with each participant classified by COVID-19 infection history. Before modeling, data were pre-processed by scaling z-scores, removing incomplete records, and harmonizing variable labels across datasets.

To address potential multicollinearity among the three standardized diffusion variables, PCA was conducted using the *prcomp* function in R. This analysis yielded three orthogonal principal components: PC1 primarily represented a global impairment in gas exchange (with negative loadings across DLNO_5s_, DLCO_5s_, and VA_5s_), PC2 reflected differences in alveolar volume relative to the gas transfer variables, and PC3 isolated DLNO_5s_-specific effects. These components were subsequently used as predictors in logistic regression models to evaluate their association with COVID-19 status.

Multiple logistic regression models were implemented, including both fixed-effects models and mixed-effects models incorporating a random intercept for the study site. Fixed-effects models examined individual predictors (DLNO_5s_, DLCO_5s_, VA_5s_), pairwise combinations, full three-variable models, and PCA-derived predictors (PC1–PC3). Mixed-effects models were estimated using the *glmer* function from the lme4 package with a binomial error distribution. Model performance was evaluated using Akaike and Bayesian Information Criteria (AIC, BIC), *p*-values, odds ratios with 95 % confidence intervals, and marginal R² values derived from *MuMIn::r.squaredGLMM*.

To quantify the relative contribution of each diffusion variable to the overall explained variance, hierarchical partitioning was performed using the *glmm.hp* function from the glmm.hp package. This technique apportioned the explained variance (ΔR²) among individual predictors in the presence of covariation.

### 3.6 Performance evaluation of DLCO_5s_ z-scores and summed DLNO_5s_ + DLCO_5s_ z-scores

Model performance was evaluated in two stages. In the first stage, we compared a ``standard'' mixed-effects logistic regression model—including only DLCO₅s z-scores as the predictor—to a ``combined'' model that incorporated the sum of DLCO_5s_ ₅s and DLNO_5s_ z-scores. Both models were fitted using the *glmer* function from the *lme4* R package (family = binomial), with a random intercept specified for study location to account for site-level variability. Predicted probabilities were calculated for each participant under both models. To derive binary classification outcomes, we identified the optimal probability threshold by maximizing Youden's J statistic (sensitivity + specificity − 1) on the urea under the receiver operating characteristic (AUROC) curve using the *pROC* package. This threshold was then applied to produce confusion matrices, from which we computed sensitivity, specificity, overall misclassification rate (i.e., [false positives + false negatives]/total), and the Matthews Correlation Coefficient (MCC)—a balanced accuracy measure that incorporates all four cells of the confusion matrix and is written as:

$$\text{MCC}=\frac{\left(TP\times TN)-(FP\times FN\right)}{\sqrt{\left(TP+FP)(TP+FN)(TN+FP)(TN+FN\right)}}$$

Where TP = true positives; TN = true negatives; FP = false positives; FN = false negatives. The interpretation of the MCC is as follows:

• MCC = +1 → perfect prediction
• MCC = 0 → random prediction
• MCC = −1 → total disagreement between prediction and observation

In the second stage, we performed a threshold-free evaluation to more comprehensively assess the improvement in classification performance. First, we re-estimated Youden's J and its 95 % confidence interval using 1000 bootstrap replicates via *ci.coords* in *pROC*. Next, we quantified the category-free Net Reclassification Improvement (NRI), defined as the net proportion of cases and controls whose predicted probabilities moved in the correct direction (i.e., upward for cases, downward for controls) when switching from the standard to the combined model. This was implemented using the nribin() function from the *nricens* package (updown = ``diff,'' cut = 0) with 10,000 bootstrap samples to derive standard errors and 95 % percentile intervals.

Finally, we computed the Integrated Discrimination Improvement (IDI), which measures the change in discrimination slope—the difference in average predicted risk between cases and controls—between the two models. The IDI and its 95 % confidence interval were estimated using 10,000 bootstrap replicates via the *boot* package.

### 3.7 The association between pulmonary diffusing capacity and dyspnoea severity

The relationship between lung function z-scores and dyspnoea severity was explored by analysing exertional breathlessness, as documented on the modified Medical Research Council (mMRC) dyspnoea scale [32]. This analysis employed polyserial correlation and proportional odds models for studies reporting exertional breathlessness data [[8], [9], [10], [11], [12], [13], [15]]. A Bayesian mixed-effects cumulative link model was utilized to evaluate the probability of progressing to higher dyspnoea categories based on z-scores, incorporating a random intercept to account for variability across studies. Pairwise comparisons between dyspnoea categories were conducted using estimated marginal means (EMMs) obtained through the *emmeans* R package, with differences quantified using posterior distributions. Results were summarized by reporting posterior medians and 95 % credible intervals for pairwise differences and their associated uncertainty. Additionally, intraclass correlation coefficients (ICCs) were estimated from the posterior distribution of variance components in the Bayesian cumulative link mixed models, representing the proportion of variability in dyspnoea categories attributable to differences between studies. Posterior medians and 95 % credible intervals were also provided for the intraclass correlation coefficient estimates.

### 3.8 R code

All R-packages used in the statistical analyses are listed in the online supplementary file. The online supplement includes the R-code to run most analyses. Contained within the supplementary file is the code to rank-order the models from best-fit to worst-fit in predicting those with previous COVID-19 compared to matched controls using the BIC and LOOIC. The difference in computational speed between BIC and LOOIC lies in their methodologies and the computational processes they require. BIC relies on a closed-form formula that evaluates model performance by combining the likelihood of the model and a penalty for the number of parameters. This formula is straightforward and deterministic, as it involves calculating the maximum likelihood estimate (MLE) and applying a simple adjustment based on the sample size and the number of model parameters [33]. The BIC is a relatively simple penalized likelihood criterion used to evaluate model fit. For a given model, BIC is calculated as:

BIC=−2·loglikelihood+k·log(n)

Where *k* is the number of parameters in the model and *n* is the sample size [33]. Computing BIC is efficient because it involves fitting the model once and extracting its log-likelihood and parameter count. Since no iterative processes or resampling steps are involved, BIC computations are efficient and fast, often completing in seconds on most computers.

In contrast, LOOIC is based on a more complex procedure involving leave-one-out cross-validation [26]. This approach evaluates model performance by iteratively removing one observation at a time from the dataset, fitting the model to the remaining data, and calculating how well the model predicts the omitted observation. This process is repeated for each data point, requiring extensive resampling and re-estimation of the model multiple times. The computational intensity of these iterative steps is further magnified for more complex models or larger datasets. As a result, calculating LOOIC in these datasets can take significantly longer—often nearly an hour—depending on the computational power available. This difference in computational speed reflects the trade-off between the simplicity of BIC’s approximation and the robustness of LOOIC’s predictive validation.

All analyses were performed using R version 4.3.2 and RStudio version 2025.05.0 (Build 496). Statistical significance was defined as *p* < 0.05 for all frequentist analyses.

## Limitations

The study has several limitations. First, the dataset is limited to White participants, which restricts the generalizability of the findings to more diverse populations. Second, the absence of pre-COVID-19 baseline pulmonary function data necessitated reliance on reference equations to estimate impairments, which may introduce biases. Third, approximately 18 % of the control group exhibited pulmonary impairments, potentially affecting the specificity and accuracy of classification metrics. Finally, the exclusion of paediatric populations limits the applicability of the findings to adults.

## Ethics Statement

Ethical approval for anonymized data collection was obtained for all original studies [8–17]. Secondary analysis of de-identified data was exempt from requiring additional Institutional Review Board (IRB) approval.

## Credit Author Statement

G.S.*Z* = Conceptualization; Methodology; Software (programming, coding); Formal analysis; Investigation; Data Curation; Writing (original draft, review and editing); Project Administration. G.B. = Resources; Investigation. T.G. = Resources; Investigation. R.W.D-N. = Resources; Investigation. M.N-F. = Resources; Investigation. L.S. = Resources; Investigation. G.I. Resources; Investigation. F.D. = Resources; Investigation. J.M. = Resources, Investigation. E.S. = Resources, Investigation; P.A. = Resources, Investigation. V.B. = Resources; Investigation.

## Declaration of Generative AI and AI-assisted Technologies in the Writing Process

During the preparation of the main manuscript, Gerald S. Zavorsky used CHAT GPT to improve sentence structure and grammar. After using this tool, Gerald S. Zavorsky reviewed and edited the content as needed and takes full responsibility for the content of the publication.

## Data Availability

Mendeley DataThe long term effects of COVID-19 on Pulmonary diffusing capacity for nitric oxide (DLNO) and carbon monoxide (DLCO) (Reference data).

## Appendix Supplementary materials

Image, application 1
