# Supplementary material for: Pulmonary diffusing capacity and dyspnoea following COVID-19: Insights from multicentre datasets
Source: Data Brief. 2025 Jul 25;62:111925. doi: 10.1016/j.dib.2025.111925 (PMC12361787; doi:10.1016/j.dib.2025.111925)
Supplement: Supplementary file 1 [file mmc1.pdf]

Supplement to the article: “Pulmonary diffusing capacity and dyspnoea following COVID-19: Insights from multicentre datasets”

[1]Gerald S. Zavorsky PhD, [2]Giovanni Barisione MD, [3]Thomas Gille MD, [4]Roberto W. Dal-Negro MD, [5,6]Marta Núñez-Fernández MD, [7,8]Leigh Seccombe PhD, [9]Gianluca Imeri MD, [9]Fabiano Di Marco MD PhD, [10]Jann Mortensen MD DMSc, [11]Elisabetta Salvioni PhD, [11]Piergiuseppe Agostoni MD PhD, [12]Vito Brusasco MD

[1]Department of Physiology and Membrane Biology, University of California Davis, Sacramento, California, United States

[2]Struttura Semplice Fisiopatologia Respiratoria, Clinica Malattie Respiratorie e Allergologia, IRCCS Ospedale Policlinico San Martino, Genoa, Italy

[3]Department of Physiology and Functional Exploration, University Hospitals of Paris Seine-Saint-Denis, HP–AP, Bobigny, France

[4]CESFAR – National Center for Respiratory Pharmacoeconomics and Pharmacoepidemiology, 37124, Verona, Italy

[5]Service of Pneumology, University Hospital Complex of Vigo, Vigo, Spain

[6]NeumoVigo, Institute of Health Research South Galicia (IISGS), 36213, Vigo, Spain

[7]The University of Sydney, Camperdown, NSW, Australia

[8]Thoracic Medicine, Concord Repatriation General Hospital, Concord, NSW, Australia

[9]Department of Health Sciences, University of Milano, ASST Papa Giovanni XXIII Hospital, Bergamo, Italy

[10]Department of Clinical Physiology and Nuclear Medicine, Rigshospitalet, Copenhagen University Hospital, Copenhagen, Denmark

[11]Centro Cardiologico Monzino IRCCS, Milan, Italy

[12]Department of Experimental Medicine, University of Genoa, Genoa, Italy.

ORCID

Gerald Stanley Zavorsky[1] 0000-0002-4473-1601
Giovanni Barisione[2] 0000-0002-2349-5646
Thomas Gille[3] 0000-0002-1577-6595
Roberto Dal Negro[4] 0000-0001-6305-0732
Marta Núñez-Fernández[5,6] 0000-0001-9088-3713
Leigh Seccombe[7,8] 0000-0001-8037-3824
Gianluca Imeri[9] 0000-0003-1139-6509
Fabiano Di Marco[9] 0000-0002-1743-0504
Jann Mortensen[10] 0000-0002-1399-8995
Elisabetta Salvioni[11] 0000-0002-8448-7551
Piergiuseppe Agostoni[11] 0000-0002-8345-6382
Vito Brusasco[12]: 0000-0001-8948-0896

Address for correspondence

Gerald S. Zavorsky, Ph.D., RRT, RPFT, FACSM
Full Professor (Adjunct Series)
Department of Physiology and Membrane Biology
University of California at Davis
Tupper Hall, Rm 4327
1275 Medical Sciences Drive
Davis, CA, 95616
gszavorsky@health.ucdavis.edu

Contents

Filtering the data = “Covid19_&_Controls.sav”, then saving the Filtered data 3

Comparing anthropometrics, and pulmonary function variables between those with previous COVID-19 and controls. Using BH procedure, controlling for multiple paired comparisons at the false discovery rate of 0.05. 9

Goodness of fit using the Bayesian Information Criterion (BIC) and Leave-One-Out Information Criterion (LOOIC) 21

Model summaries and fit; Confusion-matrix counts (TP, TN, FP, FN) at Youden’s J–optimal threshold, ranked by MCC 33

Model performance summary when adding $DLNO_{5s}$ z-scores to the best $DLCO_{5s}$-only z-scores model. 42

Agreement Analysis 46

The Area Under the Receiver Operating Characteristic Curve (AUROC) and Mathews Correlation Coefficient (MCC) 50

Polyserial correlations between the modified Medical Research Council dyspnea score (mMRC, 0 to 4), and pulmonary function test z-scores. 61

Bayesian mixed-effects proportional odds logistic regression analysis to model the relationship between various lung function z-scores and the ordinal outcome MRC_Dyspnea 70

References 72

Note: The following pages display the R programming code for various statistical analyses including figure generation from the multicentre studies located in the cloud based online repository Mendeley Data[1]. The data was obtained from previously published articles[2-9] and abstracts [10 11], and then pooled. The R programming code was created solely by Gerald S. Zavorsky with the help of GroK 3, and R Wizard (04-mini-high) from CHAT GPT.

All analyses were conducted in R v4.3.2 and RStudio 2025.05.0 (build 496)

Filtering the data = "Covid19_&_Controls.sav", then saving the Filtered data

```
# ---------------------------------------------------------------------------------------------------------------
# Load required packages
# ---------------------------------------------------------------------------------------------------------------
library(dplyr)
library(haven)
library(labelled)
library(writexl)

# ---------------------------------------------------------------------------
# STEP 1: Read SPSS file [note: the location of the data is dependent on where the user has
stored the file. As such, STEP1 will be dependent on where the user saved the file
Covid19_&_Controls.sav]
# ---------------------------------------------------------------------------
input_sav <- "C:/Users/gzavorsky/OneDrive - UC Davis Health/Desktop/Mendeley data Version 3
June 2025/Covid19_&_Controls.sav"
if (!file.exists(input_sav)) stop("Input file not found: ", input_sav)
covid_raw <- read_sav(input_sav)

# ---------------------------------------------------------------------------------------------------------------
# STEP 1b: Prepare ASCII-safe abbreviations for Study
# ---------------------------------------------------------------------------------------------------------------
study_levels <- levels(as_factor(covid_raw$Study))
study_ascii  <- iconv(study_levels, from = "UTF-8", to = "ASCII//TRANSLIT")
study_abbrev <- abbreviate(study_ascii, minlength = 20)

# ---------------------------------------------------------------------------------------------------------------
# STEP 2: Define Z-score vars for outlier screening
# ---------------------------------------------------------------------------------------------------------------
z_vars <- c(
  "TLC_Zscores", "DLCO_Zscores_ALL", "DLNO_Zscores_ALL",
  "DLCO_Zscores_GAMLSS", "DLCO_Zscores_Segmented", "DLCO_Zscores_ERS",
  "DLCO_Zscores_GLI", "DLCO_Zscores_Zavorsky", "DLCO_Zscores_Aguilaniu",
  "DLNO_Zscores_GAMLSS", "DLNO_Zscores_Segmented", "DLNO_Zscores_ERS",
  "DLNO_Zscores_Zavorsky", "DLNO_Zscores_Aguilaniu",
  "FEV1_Zscores", "FVC_Zscores"
)

# ---------------------------------------------------------------------------------------------------------------
# STEP 3: Clean & filter (drop 'Location' column)
# ---------------------------------------------------------------------------------------------------------------
covid_clean <- covid_raw %>%
  slice(1:841) %>%
  mutate(
    Study_Location = factor(
      Study_Location,
```

```
      levels = 1:8,
      labels = c(
        "Verona, Italy", "Genova, Italy", "Vigo, Spain", "Bobigny, France",
        "Sydney, Australia", "Bergamo, Italy", "Copenhagen, Denmark", "Milan, Italy"
      )
    ),
    Study   = factor(as_factor(Study), labels = study_abbrev),
    Disease = as.numeric(Disease),
    Sex     = as.numeric(Sex),
    Machine = as.numeric(Machine)
  ) %>%
  filter(
    Machine == 0,
    VA_minus_TLC   <= 0.190,
    FEV1_FVC_Ratio <= 0.95,
    !(
      Disease == 0 &
        if_any(all_of(z_vars), ~ .x < -2.5 | .x > 2.5)
    )
  )

# ------------------------------------------------------------------------------------------------------------------------
# STEP 4: Recode key vars into descriptive factors & attach labels
# ------------------------------------------------------------------------------------------------------------------------
covid_labeled <- covid_clean %>%
  mutate(
    Study = factor(
      as.numeric(as_factor(Study)),  # ensure underlying codes 1:12
      levels = 1:12,
      labels = c(
        "1. Dal Negro et al. (2022)",
        "2. Barisione & Brusasco (2023)",
        "3. Barisinoe & Brusacso (2021)",
        "4. Nunez-Fernandez (2021)",
        "5. Sese et al. (2022) [abstract] FRENCH",
        "6. Thomas Gille Unpublished",
        "7. Sese et al. (2022) ERJ [Abstract]",
        "8. Seccombe et al. (2023)",
        "9. Imeri et al. (2024)",
        "10. Lytzen et al. (2024)",
        "11. Magini et al. (2015)",
        "12. Agostoni et al. (2024)"
      )
    ),
    TLC_LLN = factor(
      TLC_LLN, levels = 1:2,
      labels = c("No Restriction", "Restriction")
    ),
    Obstruction = factor(
      Obstruction, levels = 1:2,
      labels = c("No airway obstruction", "Airway obstruction")
```

```
),
Spirometry_Restriction = factor(
  Spirometry_Restriction, levels = 1:2,
  labels = c("No restrictive spirometry pattern", "Restrictive spirometry pattern")
),
Mixed = factor(
  Mixed, levels = 1:2,
  labels = c(
    "No mixed airway obstruction and restriction",
    "Yes, both airway obstruction & restriction"
  )
),
Obstruction_or_Restriction_or_Mixed = factor(
  Obstruction_or_Restriction_or_Mixed, levels = 1:2,
  labels = c(
    "No obstruction, restriction, or mixed disease",
    "Yes, one or more of obstruction, restriction, or mixed disease"
  )
),
Any_issue = factor(
  Any_issue, levels = 1:2,
  labels = c(
    "No abnormal lung function indices",
    "Yes, at least one of: obstruction/restriction/mixed or DLCO/DLNO < LLN"
  )
),
Disease = factor(
  Disease, levels = 1:2,
  labels = c(
    "Controls, never having previous COVID-19",
    "Patients with previous COVID-19"
  )
),
Sex = factor(
  Sex, levels = 1:2,
  labels = c("females", "males")
),
Machine = factor(
  Machine, levels = 1:2,
  labels = c("Jaeger MasterScreen PFT Pro", "Hyp'Air Compact")
),
Ethnicity = factor(
  Ethnicity, levels = 1:5,
  labels = c("white", "black", "asian", "hispanic", "arab")
),
Days_BTW_Group = factor(
  Days_BTW_Group, levels = 1:5,
  labels = c(
    "no previous COVID-19",
    "17-90 days post-COVID-19",
    "91-180 days post-COVID-19",
```

```
      "181-360 days post-COVID-19",
      "370-579 days post-COVID-19"
    )
  ),
  Age_Group = factor(
    Age_Group, levels = 1:9,
    labels = c(
      "0-9 yrs", "10-19 yrs", "20-29 yrs", "30-39 yrs",
      "40-49 yrs", "50-59 yrs", "60-69 yrs", "70-79 yrs",
      "80-90 yrs"
    )
  ),
  BMI_Categories = factor(
    BMI_Categories, levels = 1:6,
    labels = c(
      "Underweight (BMI < 18.5)",
      "Normal (BMI 18.5-24.9)",
      "Overweight (BMI 25.0-29.9)",
      "Obese I (BMI 30.0-34.9)",
      "Obese II (BMI 35.0-39.9)",
      "Obese III (BMI 40.0-49.9)"
    )
  ),
  DLNO_Segmented_LLN = factor (DLNO_Segmented_LLN, levels = 1:2, labels = c("No, not below
the LLN", "Yes, below the LLN")),

  DLNO_GAMLSS_LLN = factor (DLNO_GAMLSS_LLN, levels = 1:2, labels = c("No, not below the
LLN", "Yes, below the LLN")),

  DLNO_Munkholm_LLN = factor (DLNO_Munkholm_LLN, levels = 1:2, labels = c("No, not below the
LLN", "Yes, below the LLN")),

DLCO_Segmented_LLN = factor (DLCO_Segmented_LLN, levels = 1:2, labels = c("No, not below the
LLN", "Yes, below the LLN")),

DLCO_GAMLSS_LLN = factor (DLCO_GAMLSS_LLN, levels = 1:2, labels = c("No, not below the LLN",
"Yes, below the LLN")),

DLCO_Munkholm_LLN = factor (DLCO_Munkholm_LLN, levels = 1:2, labels = c("No, not below the
LLN", "Yes, below the LLN")),

VA_Segmented_LLN = factor(VA_Segmented_LLN, levels = 1:2, labels = c("No, not below the LLN",
"Yes, below the LLN")),

VA_GAMLSS_LLN = factor( VA_GAMLSS_LLN, levels = 1:2, labels = c("No, not below the LLN", "Yes,
below the LLN")), VA_Munkholm_LLN = factor( VA_Munkholm_LLN, levels = 1:2,labels = c("No, not
below the LLN", "Yes, below the LLN")),

Combined_Zscores_Segmented_LLN = factor( Combined_Zscores_Segmented_LLN, levels = 1:2,
labels = c("No, not below the LLN", "Yes, below the LLN")),
```

```
Combined_Zscores_GAMLSS_LLN = factor( Combined_Zscores_GAMLSS_LLN, levels = 1:2,
labels = c("No, not below the LLN", "Yes, below the LLN")),

Combined_Zscores_Munkholm_LLN = factor( Combined_Zscores_Munkholm_LLN, levels = 1:2,
labels = c("No, not below the LLN", "Yes, below the LLN")),

DLNO_Zscore_Classification = factor( DLNO_Zscore_Classification, levels = 1:5, labels = c( "Normal,
z-scores between -1.645 and +1.645", "Mild Decrease, z-scores between -3.50 and -1.646", "Moderate
Decrease, z-scores between -5.00 and -3.51", "Severe Decrease, z-scores -5.01 and below",
"Increased, z-scores > +1.645")),

DLCO_Zscore_Classification = factor( DLCO_Zscore_Classification, levels = 1:5,labels = c(
"Normal, z-scores between -1.645 and +1.645", "Mild Decrease, z-scores between -3.50 and -1.646",
"Moderate Decrease, z-scores between -5.00 and -3.51", "Severe Decrease, z-scores -5.01 and
below", "Increased, z-scores > +1.645"))

 ) %>%
 set_variable_labels(
  Study                              = "Research study",
  Study_Location                     = "Study location",
  TLC_LLN                            = "TLC below LLN",
  Obstruction                        = "Airway obstruction",
  Spirometry_Restriction             = "Restrictive spirometry pattern",
  Mixed                              = "Mixed airway obstruction + restriction",
  Obstruction_or_Restriction_or_Mixed = "Obstruction/restriction/mixed present",
  Any_issue                          = "Any abnormal lung function issue",
  Disease                            = "Disease status",
  Sex                                = "Biological sex",
  Machine                            = "Measurement device",
  Ethnicity                          = "Ethnicity",
  Days_BTW_Group                     ="Days between COVID-19 groups",
  Age_Group                          = "Age group",
  BMI_Categories                     = "BMI category",
  DLNO_Segmented_LLN                 = "DLNO segmented below LLN",
  DLNO_GAMLSS_LLN                    = "DLNO GAMLSS below LLN",
  DLNO_Munkholm_LLN                  = "DLNO Munkholm below LLN",
  DLCO_Segmented_LLN                 = "DLCO segmented below LLN",
  DLCO_GAMLSS_LLN                    = "DLCO GAMLSS below LLN",
  DLCO_Munkholm_LLN                  = "DLCO Munkholm below LLN",
  VA_Segmented_LLN                   = "VA segmented below LLN",
  VA_GAMLSS_LLN                      = "VA GAMLSS below LLN",
  VA_Munkholm_LLN                    = "VA Munkholm below LLN",
  Combined_Zscores_Segmented_LLN     = "Combined Z-scores segmented below LLN",
  Combined_Zscores_GAMLSS_LLN        = "Combined Z-scores GAMLSS below LLN",
  Combined_Zscores_Munkholm_LLN      = "Combined Z-scores Munkholm below LLN",
  DLNO_Zscore_Classification         = "DLNO Z-score classification",
  DLCO_Zscore_Classification         = "DLCO Z-score classification",
  VA_minus_TLC                       = "VA - TLC difference",
  FEV1_FVC_Ratio                     = "FEV1/FVC ratio"
 )
```

```
# ------------------------------------------------------------------------------------------------------------------------
# STEP 5: Summarize disease status
# ------------------------------------------------------------------------------------------------------------------------
covid_summary <- covid_labeled %>%
  count(Disease) %>%
  rename(disease_status = Disease) %>%
  mutate(percent = round(100 * n / sum(n), 1))
print(covid_summary)
# ------------------------------------------------------------------------------------------------------------------------
# STEP 6: Export cleaned data [note: the location of where the filtered data will be saved is
dependent on where the user has stored the file. As such, STEP 6 will be dependent on where
the user saved the file Covid19_&_Controls.sav]
# ------------------------------------------------------------------------------------------------------------------------
base_dir <- "C:/Users/gzavorsky/OneDrive - UC Davis Health/Current Working
Manuscripts/COVID-19 paper"
sav_dir  <- file.path(base_dir, "SPSS Files")
xlsx_dir <- file.path(base_dir, "Excel")
ensure_dir <- function(dir) if (!dir.exists(dir)) dir.create(dir, recursive = TRUE)
ensure_dir(sav_dir); ensure_dir(xlsx_dir)

sav_path  <- file.path(sav_dir,  "Covid_19_&_Controls_Filtered.sav")
xlsx_path <- file.path(xlsx_dir, " Covid_19_&_Controls_Filtered.xlsx")

# Test write-permission
test_file <- file.path(sav_dir, ".__write_test__.txt")
ok <- tryCatch({ writeLines("ok", test_file); file.remove(test_file); TRUE }, error = function(e) FALSE)
if (!ok) stop("Cannot write to directory: ", sav_dir)
haven::write_sav(covid_labeled, sav_path)
message("SPSS file written to: ", sav_path)

covid_labeled %>%
  mutate(across(where(is.numeric), ~ round(.x, 2))) %>%
  write_xlsx(xlsx_path)
message("Excel file written to: ", xlsx_path)
```

Comparing anthropometrics, and pulmonary function variables between those with previous COVID-19 and controls. Using BH procedure, controlling for multiple paired comparisons at the false discovery rate of 0.05.

```
# 1—— Load Packages & Setup
library(dplyr)
library(haven)
library(moments)
library(future)
library(flextable)
library(ggplot2)
library(tidyr)
library(knitr)
library(purrr)
library(broom)
library(tibble)

plan(multisession, workers = min(14, parallel::detectCores() - 1))
set.seed(123)
tic <- Sys.time()

# 2—— Prepare Data
covid <- Covid19_Controls_Filtered  |>
  na.omit() |>
  mutate(across(where(haven::is.labelled), as.numeric)) |>
  slice_head(n = 644)

#3 —— Subject Counts
subject_counts <- list(
  num_diseased = sum(covid$Disease == 1, na.rm = TRUE),
  num_control  = sum(covid$Disease == 0, na.rm = TRUE)
)

cat("Actual Diseased:", subject_counts$num_diseased, "\n")
cat("Actual Controls:", subject_counts$num_control, "\n")

subject_counts_df <- data.frame(
  category = c("Diseased", "Controls"),
  expected = c(572, 72),
  actual   = c(subject_counts$num_diseased, subject_counts$num_control)
)

flextable(subject_counts_df) |>
```

```
  autofit() |>
  print()

if (
  subject_counts$num_diseased != 572 ||
  subject_counts$num_control  != 72
) {
  warning(
    "The number of diseased (", subject_counts$num_diseased,
    ") and/or controls (", subject_counts$num_control,
    ") does not match the expected values (572, 72)!"
  )
} else {
  cat("✅ Counts match expected values: 572 diseased, 72 controls\n")
}

cat("\n✅ Global filtering complete. Remaining rows:", nrow(covid), "\n")
toc <- Sys.time()
cat("Model evaluation time:", round(difftime(toc, tic, units = "mins"), 2), "minutes\n")

# 4—— BHT Skewness & Quartiles
bht_skewness <- skewness(covid$BHT, na.rm = TRUE)

bht_quartiles <- covid |>
  summarise(
    median_bht = median(BHT, na.rm = TRUE),
    q1_bht     = quantile(BHT, 0.25, na.rm = TRUE),
    q3_bht     = quantile(BHT, 0.75, na.rm = TRUE)
  )

if (abs(bht_skewness) < 0.5) {
  bht_summary <- covid |>
    summarise(
      mean_bht = mean(BHT, na.rm = TRUE),
      sd_bht   = sd(BHT, na.rm = TRUE)
    )

  cat(
    "✅ BHT appears normally distributed (|skewness| < 0.5).\n",
    "Mean:", bht_summary$mean_bht, "\n",
    "SD:  ", bht_summary$sd_bht, "\n"
  )
} else {
  cat(
    "⚠️ BHT is not normally distributed. Skewness =",
    format(bht_skewness, digits = 3), "\n"
  )
}

cat("\n📈 Skewness of BHT:\n")
```

```r
print(bht_skewness)

cat("📊 Median, Q1, and Q3 of BHT:\n")
print(bht_quartiles)

#5 —— Male Count by Disease Group
male_counts <- covid |>
  group_by(Disease) |>
  summarise(male_count = sum(Sex == 1, na.rm = TRUE), .groups = "drop")

group_counts <- covid |>
  group_by(Disease) |>
  summarise(patient_count = n(), .groups = "drop")

print(male_counts)
print(group_counts)

# 6 —— DLNO/DLCO Ratio Summary
covid <- covid |>
  rename(dlnodlco_ratio = DLNODLCO_ratio_Sealevel_Hb)

dlno_dlco_summary <- covid |>
  summarise(
    mean    = round(mean(dlnodlco_ratio, na.rm = TRUE), 3),
    sd      = round(sd(dlnodlco_ratio,   na.rm = TRUE), 3),
    median  = round(median(dlnodlco_ratio, na.rm = TRUE), 3),
    q1      = round(quantile(dlnodlco_ratio, 0.25, na.rm = TRUE), 3),
    q3      = round(quantile(dlnodlco_ratio, 0.75, na.rm = TRUE), 3),
    n       = n(),
    .by     = Disease
  )

kable(dlno_dlco_summary, caption = "DLNO/DLCO Ratio Summary by Disease", digits = 3)

# 7 —— Distribution Plot
ggplot(covid, aes(x = dlnodlco_ratio, colour = as.factor(Disease), fill = as.factor(Disease))) +
  geom_histogram(alpha = 0.4, bins = 30, position = "identity") +
  geom_density(alpha = 0.2) +
  scale_fill_brewer(palette = "Set1") +
  scale_color_brewer(palette = "Set1") +
  labs(
    title = "Distribution of DLNO/DLCO Ratio by Disease",
    x = "DLNO/DLCO Ratio",
    fill = "Disease",
    colour = "Disease"
  ) +
  theme_minimal()

# 8 —— Normality Tests
normality_results <- covid |>
  group_by(Disease) |>
```

```
  group_map(
    ~ {
      x <- .x$dlnodlco_ratio
      sw <- shapiro.test(x)
      skew <- skewness(x, na.rm = TRUE)
      kurt <- kurtosis(x, na.rm = TRUE)

      tibble(
        W        = round(sw$statistic, 3),
        p_value  = round(sw$p.value, 4),
        skew     = round(skew, 3),
        kurtosis = round(kurt, 3)
      )
    },
    .keep = TRUE
  ) |>
  bind_rows(.id = "Disease")

kable(normality_results, caption = "Shapiro-Wilk, Skewness, and Kurtosis by Disease", digits = 3)

# 9 —— Between-Group Comparison (DLNO/DLCO)
wilcox_res <- wilcox.test(dlnodlco_ratio ~ Disease, data = covid, exact = FALSE)

cat("Mann-Whitney U Test:\n",
    "W statistic:", round(wilcox_res$statistic, 3), "\n",
    "p-value:    ", round(wilcox_res$p.value, 4), "\n"
)

# 10 —— Continuous Variable Tests

  "Age", "Height", "Weight", "BMI", "Hb", "FEV1_Zscores", "FVC_Zscores",
  "FEV1_FVC_Ratio_Zscores", "TLC_Zscores", "DLCO_Zscores_Segmented",
  "DLNO_Zscores_Segmented", "VA_Zscores_Segmented"
)

test_one_var <- function(data, var, alpha = 0.05) {
  x0 <- data[[var]][data$Disease == 0]
  x1 <- data[[var]][data$Disease == 1]

  p0 <- if (sum(!is.na(x0)) >= 3) shapiro.test(x0)$p.value else NA_real_
  p1 <- if (sum(!is.na(x1)) >= 3) shapiro.test(x1)$p.value else NA_real_

  if (!is.na(p0) && !is.na(p1) && p0 > alpha && p1 > alpha) {
    tt <- t.test(x0, x1)
    tibble(
      variable   = var,
      test       = "t-test",
      statistic  = round(tt$statistic, 3),
      p_value    = round(tt$p.value, 4),
      normal_0   = TRUE,
      normal_1   = TRUE
```

```
    )
  } else {
    wt <- wilcox.test(x0, x1, exact = FALSE)
    tibble(
      variable   = var,
      test       = "wilcox",
      statistic  = round(wt$statistic, 3),
      p_value    = round(wt$p.value, 4),
      normal_0   = p0 > alpha,
      normal_1   = p1 > alpha
    )
  }
}

results <- map_dfr(variables, ~ test_one_var(covid, .x))
```

#11 —— Tidy & Flextable Output

```
test_results <- results |>
  rowwise() |>
  mutate(
    htest = list(
      if (test == "t-test") {
        t.test(
          covid[[variable]][covid$Disease == 0],
          covid[[variable]][covid$Disease == 1],
          var.equal = FALSE
        )
      } else {
        wilcox.test(
          covid[[variable]][covid$Disease == 0],
          covid[[variable]][covid$Disease == 1],
          exact = FALSE
        )
      }
    ),
    tidied = list(broom::tidy(htest))
  ) |>
  unnest(tidied, names_sep = "_") |>
  mutate(
    estimate  = round(tidied_estimate,  4),
    statistic = round(tidied_statistic, 2),
    p.value   = round(tidied_p.value,   4),
    conf.low  = round(tidied_conf.low,  3),
    conf.high = round(tidied_conf.high, 3)
  ) |>
  select(
    variable,
    method     = tidied_method,
    estimate,
    statistic,
    p.value,
```

```
    conf.low,
    conf.high
  )

flextable(test_results) |>
  set_header_labels(
    variable  = "Variable",
    method    = "Method",
    estimate  = "Estimate",
    statistic = "Stat",
    p.value   = "p-value",
    conf.low  = "CI lower",
    conf.high = "CI upper"
  ) |>
  theme_booktabs() |>
  autofit()

#12—— Proportion Comparisons
compare_prop <- function(data, condition, label) {
  summary_df <- data |>
    summarise(
      n_control       = sum(Disease == 0),
      events_control  = sum({{ condition }} & Disease == 0, na.rm = TRUE),
      n_diseased      = sum(Disease == 1),
      events_diseased = sum({{ condition }} & Disease == 1, na.rm = TRUE)
    )

  pt <- prop.test(
    x = c(summary_df$events_control, summary_df$events_diseased),
    n = c(summary_df$n_control, summary_df$n_diseased),
    correct = FALSE
  )

  tibble(
    condition     = label,
    n_control     = summary_df$n_control,
    prop_control  = round(summary_df$events_control / summary_df$n_control, 3),
    n_diseased    = summary_df$n_diseased,
    prop_diseased = round(summary_df$events_diseased / summary_df$n_diseased, 3),
    difference    = round((summary_df$events_diseased / summary_df$n_diseased) -
                          (summary_df$events_control / summary_df$n_control), 3),
    statistic     = round(pt$statistic, 2),
    p_value       = round(pt$p.value, 4),
    ci_lower      = round(pt$conf.int[1], 3),
    ci_upper      = round(pt$conf.int[2], 3)
  )
}
prop_results <- bind_rows(
  compare_prop(covid, FEV1_FVC_Ratio_Zscores < -1.645 & FVC_Zscores > -1.645, "Airway
obstruction"),
  compare_prop(covid, TLC_Zscores < -1.645, "Restriction"),
```

```
  compare_prop(covid, DLNO_Zscores_Segmented < -1.645, "Diffusion impairment DLNO"),
  compare_prop(covid, DLCO_Zscores_Segmented < -1.645, "Diffusion impairment DLCO"),
  compare_prop(
    covid,
    (FEV1_FVC_Ratio_Zscores < -1.645 & FVC_Zscores > -1.645) |
      (TLC_Zscores < -1.645) |
      (DLNO_Zscores_Segmented < -1.645) |
      (DLCO_Zscores_Segmented < -1.645),
    "Any impairment"
  )
)

flextable(prop_results) |>
  set_header_labels(
    condition     = "Condition",
    n_control     = "n (Ctrl)",
    prop_control  = "Prop (Ctrl)",
    n_diseased    = "n (COVID)",
    prop_diseased = "Prop (COVID)",
    difference    = "Diff",
    statistic     = "Stat",
    p_value       = "p-value",
    ci_lower      = "CI lower",
    ci_upper      = "CI upper"
  ) |>
  theme_booktabs() |>
  autofit()

# 13—— Combine & Adjust for Multiple Testing
cat_results <- prop_results |>
  rename(
    variable  = condition,
    estimate  = difference,
    p.value   = p_value,
    conf.low  = ci_lower,
    conf.high = ci_upper
  ) |>
  mutate(method = "prop.test") |>
  select(variable, method, estimate, statistic, p.value, conf.low, conf.high)

combined_results <- bind_rows(test_results, cat_results) |>
  mutate(
    p.adj  = round(p.adjust(p.value, method = "BH"), 4),
    fdr_sig = p.adj < 0.05
  ) |>
  arrange(p.adj)

flextable(combined_results) |>
set_header_labels(
variable  = "Variable/Condition",
```

```
method    = "Test", estimate  = "Estimate", statistic = "Stat", p.value   = "p-value", p.adj  = "p.adj (BH)",
conf.low  = "CI lower", conf.high = "CI upper", fdr_sig   = "FDR < 0.05" ) |>
theme_booktabs() |>
autofit()

#-----------------------------------------------------------------------------------------------------------------
#Summary
#-----------------------------------------------------------------------------------------------------------------

# 1 —— Libraries
library(dplyr)
library(tidyr)
library(purrr)
library(tibble)
library(broom)
library(writexl)

# 2 —— Helper: format sex row

summarize_sex <- function(data) {
  summarize_group <- function(df) {
    male   <- sum(df$Sex == 1, na.rm = TRUE)
    female <- sum(df$Sex == 0, na.rm = TRUE)
    total  <- male + female
    pct_m  <- round(100 * male / total)
    sprintf("%dM, %dF (%d%% M)", male, female, pct_m)
  }
  data |>
    group_split(Disease) |>
    map_chr(summarize_group)
}

#3
```

```
summarize_binary <- function(var) {
  covid |>
    group_by(Disease) |>
    summarise(
      yes = sum(.data[[var]] == 1, na.rm = TRUE),
      total = n(),
      .groups = "drop"
    ) |>
    transmute(
      summary = sprintf("%d (%d%%)", yes, round(100 * yes / total))
    ) |>
    pull(summary)
}
```

#6 —— Define continuous variable tests

```
test_one_var <- function(data, var, alpha = 0.05) {
  x0 <- data[[var]][data$Disease == 0]
  x1 <- data[[var]][data$Disease == 1]

  p0 <- if (sum(!is.na(x0)) >= 3) shapiro.test(x0)$p.value else NA_real_
  p1 <- if (sum(!is.na(x1)) >= 3) shapiro.test(x1)$p.value else NA_real_

  if (!is.na(p0) && !is.na(p1) && p0 > alpha && p1 > alpha) {
    test_res <- tryCatch(t.test(x0, x1), error = function(e) NULL)
    method <- "t-test"
  } else {
    test_res <- tryCatch(wilcox.test(x0, x1, exact = FALSE), error = function(e) NULL)
    method <- "wilcox"
  }

  if (is.null(test_res)) {
    return(tibble(
      variable  = var,
      method    = method,
      estimate  = NA_real_,
      statistic = NA_real_,
      p.value   = NA_real_,
      conf.low  = NA_real_,
      conf.high = NA_real_
    ))
  }
  tidy_res <- broom::tidy(test_res)

  tibble(
    variable  = var,
    method    = method,
    estimate  = if ("estimate" %in% names(tidy_res)) tidy_res$estimate else NA_real_,
    statistic = tidy_res$statistic,
    p.value   = tidy_res$p.value,
    conf.low  = if ("conf.low" %in% names(tidy_res)) tidy_res$conf.low else NA_real_,
    conf.high = if ("conf.high" %in% names(tidy_res)) tidy_res$conf.high else NA_real_)}
```

# 7—— Binary variable tests (proportions)

```
test_prop <- function(data, var) {
  summary <- data |>
    summarise(
      n_control       = sum(Disease == 0),
      events_control  = sum(.data[[var]] == 1 & Disease == 0, na.rm = TRUE),
      n_diseased      = sum(Disease == 1),
      events_diseased = sum(.data[[var]] == 1 & Disease == 1, na.rm = TRUE)
    )

  pt <- prop.test(
    x = c(summary$events_control, summary$events_diseased),
    n = c(summary$n_control, summary$n_diseased),
    correct = FALSE
  )

  broom::tidy(pt) |>
    mutate(
      variable  = var,
      method    = "prop.test",
      estimate  = pt$estimate[[2]] - pt$estimate[[1]],
      conf.low  = pt$conf.int[1],
      conf.high = pt$conf.int[2]
    ) |>
    select(variable, method, estimate, statistic, p.value, conf.low, conf.high)
}
```

# 8 —— Load and prepare data

```
summary_table <- tibble(
  variable = c(  # <- key used for joining
    "Sex", "Age", "Height", "Weight", "BMI",
    "Hb", "FEV1_Zscores", "FVC_Zscores", "FEV1_FVC_Ratio_Zscores",
    "TLC_Zscores", "DLNO_Zscores_Segmented", "DLCO_Zscores_Segmented",
"VA_Zscores_Segmented",
    "Obstruction", "TLC_LLN", "DLNO_Segmented_LLN", "DLCO_Segmented_LLN", "Any_issue"
  ),
  Variable = c(  # <- display labels
    "Sex (number)", "Age (years)", "Height (cm)", "Weight (kg)", "BMI (kg/m²)",
    "Hb (g/dL)", "FEV1 z-scores", "FVC z-scores", "FEV1/FVC z-scores",
    "TLC z-scores", "DLNO z-scores", "DLCO z-scores", "VA z-scores",
    "Obstruction", "Restriction", "DLNO LLN", "DLCO LLN", "Any impairment"
  ),
  Controls = c(
    sex_summary[1],
    summarize_group(covid, "Age")[1],
    summarize_group(covid, "Height")[1],
    summarize_group(covid, "Weight", 1, 1, 0)[1],
    summarize_group(covid, "BMI", 1, 1, 0)[1],
    summarize_group(covid, "Hb", 1, 1, 0)[1],
    summarize_group(covid, "FEV1_Zscores", 2, 2, 2)[1],
```

```
    summarize_group(covid, "FVC_Zscores", 2, 2, 2)[1],
    summarize_group(covid, "FEV1_FVC_Ratio_Zscores", 2, 2, 2)[1],
    summarize_group(covid, "TLC_Zscores", 2, 2, 2)[1],
    summarize_group(covid, "DLNO_Zscores_Segmented", 2, 2, 2)[1],
    summarize_group(covid, "DLCO_Zscores_Segmented", 2, 2, 2)[1],
    summarize_group(covid, "VA_Zscores_Segmented", 2, 2, 2)[1],
    summarize_binary("Obstruction")[1],
    summarize_binary("TLC_LLN")[1],
    summarize_binary("DLNO_Segmented_LLN")[1],
    summarize_binary("DLCO_Segmented_LLN")[1],
    summarize_binary("Any_issue")[1]
  ),
  `Patients with previous COVID-19` = c(
    sex_summary[2],
    summarize_group(covid, "Age")[2],
    summarize_group(covid, "Height")[2],
    summarize_group(covid, "Weight", 1, 1, 0)[2],
    summarize_group(covid, "BMI", 1, 1, 0)[2],
    summarize_group(covid, "Hb", 1, 1, 0)[2],
    summarize_group(covid, "FEV1_Zscores", 2, 2, 2)[2],
    summarize_group(covid, "FVC_Zscores", 2, 2, 2)[2],
    summarize_group(covid, "FEV1_FVC_Ratio_Zscores", 2, 2, 2)[2],
    summarize_group(covid, "TLC_Zscores", 2, 2, 2)[2],
    summarize_group(covid, "DLNO_Zscores_Segmented", 2, 2, 2)[2],
    summarize_group(covid, "DLCO_Zscores_Segmented", 2, 2, 2)[2],
    summarize_group(covid, "VA_Zscores_Segmented", 2, 2, 2)[2],
    summarize_binary("Obstruction")[2],
    summarize_binary("TLC_LLN")[2],
    summarize_binary("DLNO_Segmented_LLN")[2],
    summarize_binary("DLCO_Segmented_LLN")[2],
    summarize_binary("Any_issue")[2]
  )
)
# 9—— Run tests & adjust p-values
continuous_vars <- c(
  "Age", "Height", "Weight", "BMI", "Hb",
  "FEV1_Zscores", "FVC_Zscores", "FEV1_FVC_Ratio_Zscores",
  "TLC_Zscores", "DLNO_Zscores_Segmented", "DLCO_Zscores_Segmented",
  "VA_Zscores_Segmented"
)

binary_vars <- c(
  "Obstruction", "TLC_LLN",
  "DLNO_Segmented_LLN", "DLCO_Segmented_LLN",
  "Any_issue"
)
test_results <- map_dfr(continuous_vars, ~ test_one_var(covid, .x))
cat_results  <- map_dfr(binary_vars, ~ test_prop(covid, .x))

combined_tests <- bind_rows(test_results, cat_results) |>
  mutate(
```

```
    p.adj   = round(p.adjust(p.value, method = "BH"), 4),
    fdr_sig = ifelse(p.adj < 0.05, "Yes", "No")
  )
```

#10 —— Merge & Save [note: the location of where the results are saved data is dependent on where the user will store the file]

```
final_table <- summary_table |>
  left_join(combined_tests, by = "variable") |>
  relocate(method, .after = `Patients with previous COVID-19`) |>
  relocate(p.value, p.adj, fdr_sig, estimate, statistic, conf.low, conf.high, .after = method)

write_xlsx(
  final_table,
  path = file.path(
    "C:/Users/gzavorsky/OneDrive - UC Davis Health/Current Working Manuscripts/COVID-19 paper",
    "Table1x.xlsx"
  )
)
```

Goodness of fit using the Bayesian Information Criterion (BIC) and Leave-One-Out Information Criterion (LOOIC)

#1 Set seed for reproducibility
set.seed(123)

#2 Define base directory for input dataset [note: the location of the data is dependent on where the user has stored the file. As such, STEP2 will be dependent on where the user saved the file Covid19_&_Controls_Filtered.sav]

base_path <- "C:/Users/gzavorsky/OneDrive - UC Davis Health/Desktop/Mendeley data Version 3 June 2025"

#3 Define output directory for results and figures [note: the location where the results are saved is dependent on where the user will save the results]

output_path <- "C:/Users/gzavorsky/OneDrive - UC Davis Health/Current Working Manuscripts/COVID-19 paper"

# 4 Load required packages
library(tidyverse)
library(conflicted)
library(AICcmodavg)
library(boot)
library(tictoc)
library(brms)
library(blme)
library(caret)
library(glmnet)
library(haven)
library(influence.ME)
library(lme4)
library(lmtest)
library(loo)
library(Matrix)
library(openxlsx)
library(parallel)
library(parallelly)
library(performance)
library(pROC)
library(rcompanion)

# 5 Resolve namespace conflicts
conflict_scout()
conflicts_prefer(dplyr::filter)
conflicts_prefer(dplyr::lag)

# 6 Start timing
tic("Model evaluation time")

```
#------------------------------------------------------------------------
BIC Evaluation
#------------------------------------------------------------------------

#7 Load dataset
dataset_file <- file.path(base_path, "Covid19_&_Controls_Filtered.sav")
if (!file.exists(dataset_file)) {
  stop("Dataset file not found at: ", dataset_file, "\nPlease check the file path or name.")
}
Covid19_Controls_Filtered <- haven::read_sav(dataset_file)

#8 Prepare data
covid <- Covid19_Controls_Filtered |>
  filter(Machine == 0) |>         # keep only Machine == 0
  slice_head(n = 644) |>          # Use n = 644
  mutate(
    study = as_factor(Study)
    # Disease remains as-is (0 & 1)
  )

#9  Parallel setup
num_cores <- availableCores(logical = FALSE) - 2
cl <- makeCluster(num_cores)
message("Using ", num_cores, " physical cores for parallel processing.")

#10 Fit all models with error handling
fit_glmer <- function(formula, data, name) {
  tryCatch({
    glmer(
      formula = formula,
      data = data,
      family = binomial,
      control = glmerControl(optimizer = "bobyqa", optCtrl = list(maxfun = 2e6), tolPwrss = 1e-4),
      nAGQ = 10
    )
  }, error = function(e) {
    warning("Model ", name, " failed: ", e$message)
    return(NULL)
  })
}

m.Zscores.TLC.mixed <- fit_glmer(Disease ~ TLC_Zscores + (1 | Study_Location), covid, "TLC")
m.Zscores.Ratio.mixed <- fit_glmer(Disease ~ FEV1_FVC_Ratio_Zscores + (1 | Study_Location),
covid, "Ratio")
m.Zscores.FVC.mixed <- fit_glmer(Disease ~ FVC_Zscores + (1 | Study_Location), covid, "FVC")
m.Zscores.FEV1.mixed <- fit_glmer(Disease ~ FEV1_Zscores + (1 | Study_Location), covid, "FEV1")
m.Zscores.VA.GLI.mixed <- fit_glmer(Disease ~ VA_Zscores_GLI + (1 | Study_Location), covid,
"VA_GLI")
m.Zscores.VA.Segmented.mixed <- fit_glmer(Disease ~ VA_Zscores_Segmented + (1 |
Study_Location), covid, "VA_Segmented")
```

```
m.Zscores.VA.GAMLSS.mixed <- fit_glmer(Disease ~ VA_Zscores_GAMLSS + (1 | Study_Location),
covid, "VA_GAMLSS")
m.Zscores.VA.Munkholm.mixed <- fit_glmer(Disease ~ VA_Zscores_Munkholm + (1 | Study_Location),
covid, "VA_Munkholm")
m.Zscores.DLCO.GLI.mixed <- fit_glmer(Disease ~ DLCO_Zscores_GLI + (1 | Study_Location), covid,
"DLCO_GLI")
m.Zscores.DLCO.Seg.mixed <- fit_glmer(Disease ~ DLCO_Zscores_Segmented + (1 |
Study_Location), covid, "DLCO_Segmented")
m.Zscores.DLCO.GAM.mixed <- fit_glmer(Disease ~ DLCO_Zscores_GAMLSS + (1 | Study_Location),
covid, "DLCO_GAMLSS")
m.Zscores.DLCO.Munk.mixed <- fit_glmer(Disease ~ DLCO_Zscores_Munkholm + (1 |
Study_Location), covid, "DLCO_Munkholm")
m.Zscores.DLNO.Seg.mixed <- fit_glmer(Disease ~ DLNO_Zscores_Segmented + (1 |
Study_Location), covid,
"DLNO_Segmented")
m.Zscores.DLNO.GAM.mixed <- fit_glmer(Disease ~ DLNO_Zscores_GAMLSS + (1 | Study_Location),
covid, "DLNO_GAMLSS")
m.Zscores.DLNO.Munk.mixed <- fit_glmer(Disease ~ DLNO_Zscores_Munkholm + (1 |
Study_Location), covid, "DLNO_Munkholm")
m.Zscores.Combined.Seg.mixed <- fit_glmer(Disease ~ Combined_Zscores_Segmented + (1 |
Study_Location), covid, "Combined_Segmented")
m.Zscores.Combined.GAM.mixed <- fit_glmer(Disease ~ Combined_Zscores_GAMLSS + (1 |
Study_Location), covid, "Combined_GAMLSS")
m.Zscores.Combined.Munk.mixed <- fit_glmer(Disease ~ Combined_Zscores_Munkholm + (1 |
Study_Location), covid, "Combined_Munkholm")
```

# 11 Define named model list

```
Cand.models <- list(
  "TLC Z-scores, GLI equations, (Hall et al. 2021)" = m.Zscores.TLC.mixed,
  "FEV1/FVC Z-scores, GLI equations, (Quanjer et al. 2012)" = m.Zscores.Ratio.mixed,
  "FVC Z-scores, GLI equations, (Quanjer et al. 2012)" = m.Zscores.FVC.mixed,
  "FEV1 Z-scores, GLI equations, (Quanjer et al. 2012)" = m.Zscores.FEV1.mixed,
  "VA Z-scores, GLI equations (Stanojevic et al. 2017)" = m.Zscores.VA.GLI.mixed,
  "VA Z-scores, SLR, (Zavorsky & Cao 2022)" = m.Zscores.VA.Segmented.mixed,
  "VA Z-scores, GAMLSS, (Zavorsky & Cao 2022)" = m.Zscores.VA.GAMLSS.mixed,
  "VA Z-scores, Munkholm et al. (2018)" = m.Zscores.VA.Munkholm.mixed,
  "DLCO Z-scores, GLI equations, (Stanojevic et al. 2017)" = m.Zscores.DLCO.GLI.mixed,
  "DLCO Z-scores, SLR, (Zavorsky & Cao 2022)" = m.Zscores.DLCO.Seg.mixed,
  "DLCO Z-scores, GAMLSS, (Zavorsky & Cao 2022)" = m.Zscores.DLCO.GAM.mixed,
  "DLCO Z-scores, Munkholm et al. (2018)" = m.Zscores.DLCO.Munk.mixed,
  "DLNO Z-scores, SLR, (Zavorsky & Cao 2022)" = m.Zscores.DLNO.Seg.mixed,
  "DLNO Z-scores, GAMLSS, (Zavorsky & Cao 2022)" = m.Zscores.DLNO.GAM.mixed,
  "DLNO Z-scores, Munkholm et al. (2018)" = m.Zscores.DLNO.Munk.mixed,
  "Combined Z-scores, SLR, (Zavorsky & Cao 2022)" = m.Zscores.Combined.Seg.mixed,
  "Combined Z-scores, GAMLSS, (Zavorsky & Cao 2022)" = m.Zscores.Combined.GAM.mixed,
  "Combined Z-scores, Munkholm et al. (2018)" = m.Zscores.Combined.Munk.mixed
)
```

# 12 Remove any NULL models (failed fits)

```
Cand.models <- Cand.models[!sapply(Cand.models, is.null)]
```

```
# 13 Parallel BIC calculations
clusterExport(cl, varlist = c("Cand.models", "BIC", "logLik", "covid"))
bic_values <- unlist(parLapply(cl, Cand.models, BIC))
min_bic <- min(bic_values)
delta_bic <- bic_values - min_bic
bic_weights <- exp(-0.5 * delta_bic) / sum(exp(-0.5 * delta_bic))
log_likelihood <- unlist(parLapply(cl, Cand.models, logLik))

#14 Build table
model_comparison <- data.frame(
  Model = names(bic_values),
  BIC = round(bic_values, 2),
  Delta_BIC = round(delta_bic, 2),
  BIC_Weight = round(bic_weights, 3),
  Cumulative_Weight = cumsum(round(bic_weights, 3)),
  Log_Likelihood = round(as.numeric(log_likelihood), 2)
)
model_comparison <- model_comparison[order(model_comparison$BIC), ]
model_comparison$Cumulative_Weight <- cumsum(model_comparison$BIC_Weight)
print(model_comparison, row.names = FALSE)

#15 Evidence ratio
evidence_ratio <- exp((model_comparison$BIC[2] - model_comparison$BIC[1]) / 2)
print(evidence_ratio)

#16  Export  [note: the location where the results are saved is dependent on where the user will
save the results]
stopCluster(cl)
bic_file <- "C:/Users/gzavorsky/OneDrive - UC Davis Health/Current Working
Manuscripts/COVID-19 paper/models_BIC1.xlsx"

# 17 Ensure directory exists
bic_dir <- dirname(bic_file)
if (!dir.exists(bic_dir)) {
  dir.create(bic_dir, recursive = TRUE)
  cat("Created directory:", bic_dir, "\n")
}
write.xlsx(model_comparison, file = bic_file, rowNames = FALSE)
cat("
```

```
# 2 Prepare data for plotting
plot_data <- data.frame(
  Model = names(delta_bic),
  BIC_Difference = delta_bic
)
plot_data <- plot_data[order(plot_data$BIC_Difference), ]
plot_data$Model <- factor(
  paste0(seq_along(plot_data$Model), ". ", plot_data$Model),
  levels = rev(paste0(seq_along(plot_data$Model), ". ", plot_data$Model))
)

#3 Dynamic x-axis limit
max_bic_diff <- max(plot_data$BIC_Difference) + 5

#4  Plot
bic_plot <- ggplot(plot_data, aes(x = BIC_Difference, y = Model)) +
  geom_rect(aes(xmin = 0.1, xmax = 2.29, ymin = -Inf, ymax = Inf), fill = "red", alpha = 0.1) +
  geom_rect(aes(xmin = 2.3, xmax = 5.99, ymin = -Inf, ymax = Inf), fill = "yellow", alpha = 0.1) +
  geom_rect(aes(xmin = 6.0, xmax = 9.29, ymin = -Inf, ymax = Inf), fill = "green", alpha = 0.1) +
  geom_rect(aes(xmin = 9.3, xmax = max_bic_diff, ymin = -Inf, ymax = Inf), fill = "purple", alpha = 0.1) +
  geom_segment(aes(x = 0, xend = BIC_Difference, y = Model, yend = Model),
               color = "black", linetype = "longdash", size = 0.8) +
  geom_point(size = 6, shape = 21, fill = "white", color = "black", stroke = 1.2) +
  scale_x_continuous(
    limits = c(0, max_bic_diff),
    breaks = seq(0, max_bic_diff, by = 5),
    minor_breaks = seq(0, max_bic_diff, by = 2.5),
    expand = c(0, 0)
  ) +
  labs(
    x = paste0("Difference in BIC compared to the best model\n(best model = ",
               names(bic_values)[which.min(bic_values)], ")"),
    y = "M O D E L S"
  ) +
  theme_minimal(base_size = 18) +
  theme(
    plot.background = element_rect(fill = "white", color = NA),
    panel.background = element_rect(fill = "white", color = NA),
    axis.ticks.length = unit(0.3, "cm"),
    axis.ticks = element_line(size = 0.8),
    axis.ticks.x = element_line(color = "black"),
    axis.ticks.y = element_line(color = "black"),
    axis.text.x = element_text(size = 18),
    axis.text.y = element_text(size = 16, hjust = 0),  # Slightly smaller to avoid overlap
    axis.title.x = element_text(size = 18, face = "bold"),
    axis.title.y = element_text(size = 18, face = "bold", margin = margin(r = 10))
  )

#5 Save plot
bic_plot_file <- file.path(output_path, "Figures", "FigureBIC.tiff")
bic_plot_dir <- dirname(bic_plot_file)
```

```
if (!dir.exists(bic_plot_dir)) {
  dir.create(bic_plot_dir, recursive = TRUE)
  cat("Created directory:", bic_plot_dir, "\n")
}
ggsave(
  filename = bic_plot_file,
  plot = bic_plot,
  device = "tiff",
  compression = "lzw",
  width = 19,
  height = 12,
  dpi = 600
)
cat("✅ BIC plot saved to:\n", bic_plot_file, "\n")
```

#——————————————————————————————————————
# LOOIC Evaluation for Multiple brms Models [Note: Execution time will vary with hardware. On a test machine equipped with a 12th Gen Intel Core i9-12950HX (2.5 GHz core speed, 1.0 GHz bus speed, 16 cores/24 threads) and 128 GB of RAM, the full LOOIC analysis was completed in 27-29 minutes. Expect runtimes between 15 and 50 minutes depending on your system's CPU generation, core count, clock speed, and memory.]
#——————————————————————————————————————

# 1) Load core libraries

```
library(conflicted)
library(dplyr)
library(tidyr)
library(brms)
library(lme4)
library(AICcmodavg)
library(Matrix)
library(caret)
library(purrr)
library(lattice)
library(boot)
library(tibble)
```

#2) Resolve conflicts

```
conflict_prefer("filter", "dplyr")
conflict_prefer("lag", "dplyr")
conflict_prefer("expand", "tidyr")
conflict_prefer("pack", "tidyr")
conflict_prefer("unpack", "tidyr")
conflict_prefer("ar", "brms")
conflict_prefer("ngrps", "brms")
conflict_prefer("checkConv", "lme4")
conflict_prefer("lift", "caret")
conflict_prefer("melanoma", "boot")
```

```
# 3) Start timing
tic("Model evaluation time")

# 4) Prepare data
covid <- Covid19_Controls_Filtered %>%
  filter(Machine == 0) %>%          # keep only Machine == 0
  slice_head(n = 644) %>%
   mutate(
    study = as_factor(Study)        # forcats::as_factor()
  )

# 5) Set priors & control
priors      <- c(
  prior(normal(0, 0.5), class = b),
  prior(exponential(1),     class = sd)
)
control_list <- list(
  adapt_delta    = 0.99,
  max_treedepth = 12
)
# 6) Define formulas & labels
formulas <- list(
  Disease ~ TLC_Zscores                + (1 | Study_Location),
  Disease ~ FEV1_FVC_Ratio_Zscores     + (1 | Study_Location),
  Disease ~ FVC_Zscores                + (1 | Study_Location),
  Disease ~ FEV1_Zscores               + (1 | Study_Location),

  Disease ~ VA_Zscores_GLI             + (1 | Study_Location),
  Disease ~ VA_Zscores_Segmented       + (1 | Study_Location),
  Disease ~ VA_Zscores_GAMLSS          + (1 | Study_Location),
  Disease ~ VA_Zscores_Munkholm        + (1 | Study_Location),

  Disease ~ DLCO_Zscores_GLI           + (1 | Study_Location),
  Disease ~ DLCO_Zscores_Segmented     + (1 | Study_Location),
  Disease ~ DLCO_Zscores_GAMLSS        + (1 | Study_Location),
  Disease ~ DLCO_Zscores_Munkholm      + (1 | Study_Location),

  Disease ~ DLNO_Zscores_Segmented     + (1 | Study_Location),
  Disease ~ DLNO_Zscores_GAMLSS        + (1 | Study_Location),
  Disease ~ DLNO_Zscores_Munkholm      + (1 | Study_Location),

  Disease ~ Combined_Zscores_Segmented + (1 | Study_Location),
  Disease ~ Combined_Zscores_GAMLSS    + (1 | Study_Location),
  Disease ~ Combined_Zscores_Munkholm  + (1 | Study_Location)
)

labels <- c(
  "TLC Z-scores, GLI equations, (Hall et al. 2021)",
  "FEV₁/FVC Z-scores, GLI equations, (Quanjer et al. 2012)",
  "FVC Z-scores, GLI equations, (Quanjer et al. 2012)",
  "FEV₁ Z-scores, GLI equations, (Quanjer et al. 2012)",
```

```
  "VA Z-scores, GLI equations (Stanojevic et al. 2017)",
  "VA Z-scores, SLR, (Zavorsky & Cao 2022)",
  "VA Z-scores, GAMLSS, (Zavorsky & Cao 2022)",
  "VA Z-scores, Munkholm et al. (2018)",

  "DLCO Z-scores, GLI equations, (Stanojevic et al. 2017)",
  "DLCO Z-scores, SLR, (Zavorsky & Cao 2022)",
  "DLCO Z-scores, GAMLSS, (Zavorsky & Cao 2022)",
  "DLCO Z-scores, Munkholm et al. (2018)",
  "DLNO Z-scores, SLR, (Zavorsky & Cao 2022)",
  "DLNO Z-scores, GAMLSS, (Zavorsky & Cao 2022)",
  "DLNO Z-scores, Munkholm et al. (2018)",
  "Combined Z-scores, SLR, (Zavorsky & Cao 2022)",
  "Combined Z-scores, GAMLSS, (Zavorsky & Cao 2022)",
  "Combined Z-scores, Munkholm et al. (2018)"
)
stopifnot(length(formulas) == length(labels))

# 7) Fit all models programmatically
models <- map2(
  formulas,
  labels,
  ~ brm(
    formula   = .x,
    data      = covid,
    family    = bernoulli(),
    prior     = priors,
    chains    = 4,
    cores     = 14,
    iter      = 4000,
    warmup    = 2000,
    control   = control_list,
    save_pars = save_pars(all = TRUE)
  )
)
names(models) <- labels

# 8) Compute LOOIC & diagnostics
loo_results <- map(
  models,
  ~ loo::loo(.x, save_psis = FALSE, reloo = TRUE)
)
looic_vals  <- map_dbl(loo_results, ~ .x$estimates["looic", "Estimate"])
delta_looic <- looic_vals - min(looic_vals)
weights     <- exp(-0.5 * delta_looic)
weights     <- weights / sum(weights)
log_liks <- map_dbl(
  models,
  ~ mean(rowSums(log_lik(.x)))
)
```

```
# 9) Build ranked comparison table
model_comparison <- tibble::tibble(
  Model            = names(looic_vals),
  LOOIC            = round(looic_vals,    2),
  Delta_LOOIC      = round(delta_looic,   2),
  LOOIC_Weight     = round(weights,       3),
  Cumulative_Weight = cumsum(round(weights, 3)),
  Log_Likelihood   = round(log_liks,      2)
) %>%
  arrange(LOOIC) %>%  # Sort by LOOIC first
  mutate(Cumulative_Weight = cumsum(LOOIC_Weight))
```

# 10) Save & display [note: the location of where the filtered data will be saved is dependent on where the user has stored the file. As such, STEP 10 will be dependent on where the user saved the file Covid19_&_Controls.sav]

```
output_path <- file.path(
  "C:/Users/gzavorsky/OneDrive - UC Davis Health",
  "Current Working Manuscripts",
  "COVID-19 paper",
  "models_LOOICx.xlsx"
)
openxlsx::write.xlsx(
  x        = model_comparison,
  file     = output_path,
  rowNames  = FALSE
)

cat("
```

```
library(conflicted)
conflict_prefer("filter", "dplyr")
conflict_prefer("lag", "dplyr")
conflict_prefer("expand", "tidyr")
conflict_prefer("pack", "tidyr")
conflict_prefer("unpack", "tidyr")

#2 Define input files [note: the location of the data is dependent on where the user has stored the files.]

bic_file <- "C:/Users/gzavorsky/OneDrive - UC Davis Health/Current Working Manuscripts/COVID-19 paper/models_BIC1.xlsx"
looic_file <- "C:/Users/gzavorsky/OneDrive - UC Davis Health/Current Working Manuscripts/COVID-19 paper/models_LOOICx.xlsx"

# Check if files exist
if (!file.exists(bic_file)) stop("Error: bic_file not found at ", bic_file)
if (!file.exists(looic_file)) stop("Error: looic_file not found at ", looic_file)

# Read data
bic_df <- read.xlsx(bic_file)
looic_df <- read.xlsx(looic_file)

#3 Inspect unique model names
cat("Unique models in bic_df:\n")
print(unique(bic_df$Model))
cat("Unique models in looic_df:\n")
print(unique(looic_df$Model))

#4 Merge and rank
combined_df <- merge(
  bic_df[, c("Model", "Delta_BIC")],
  looic_df[, c("Model", "Delta_LOOIC")],
  by = "Model",
  all = TRUE
)
combined_df <- combined_df[order(combined_df$Delta_BIC), ]
combined_df$rank <- seq_len(nrow(combined_df))

#5 Print combined_df to check merge results
print("Combined DataFrame:")
print(combined_df)

#6 Identify mismatches or missing values
if (any(is.na(combined_df$Delta_BIC)) || any(is.na(combined_df$Delta_LOOIC))) {
  cat("Warning: NA values found in Delta_BIC or Delta_LOOIC. Check the following rows:\n")
  print(combined_df[is.na(combined_df$Delta_BIC) | is.na(combined_df$Delta_LOOIC), ])
}
#7 Build labels with Unicode subscripts
combined_df$model_label <- paste0(combined_df$rank, ". ", combined_df$Model)
```

```
combined_df$model_label <- gsub("FEV1/FVC", "FEV\u2081/FVC", combined_df$model_label, fixed =
TRUE)
combined_df$model_label <- gsub("FEV1 ", "FEV\u2081 ", combined_df$model_label, fixed = TRUE)
combined_df$model_label <- factor(
  combined_df$model_label,
  levels = rev(combined_df$model_label)
)
#8 Pivot to long format
plot_df <- combined_df %>%
  pivot_longer(
    cols = c(Delta_BIC, Delta_LOOIC),
    names_to = "metric",
    values_to = "delta"
  ) %>%
  mutate(
    metric_label = ifelse(metric == "Delta_BIC", "ΔBIC", "ΔLOOIC"),
    metric_label = factor(metric_label, levels = c("ΔBIC", "ΔLOOIC"))
  ) %>%
  filter(!is.na(delta))  # Remove rows with NA delta values to avoid plotting issues

#9 Dynamic x-axis limit
max_delta <- max(plot_df$delta, na.rm = TRUE) + 5

#10 Build plot
final_plot <- ggplot(combined_df, aes(y = model_label)) +
  geom_rect(aes(xmin = 0.1, xmax = 2.29, ymin = -Inf, ymax = Inf), fill = "red", alpha = 0.1) +
  geom_rect(aes(xmin = 2.3, xmax = 5.99, ymin = -Inf, ymax = Inf), fill = "yellow", alpha = 0.1) +
  geom_rect(aes(xmin = 6.0, xmax = 9.29, ymin = -Inf, ymax = Inf), fill = "green", alpha = 0.1) +
  geom_rect(aes(xmin = 9.3, xmax = max_delta, ymin = -Inf, ymax = Inf), fill = "purple", alpha = 0.1) +
  geom_segment(
    data = plot_df,
    aes(x = 0, xend = delta, y = model_label, yend = model_label, group = metric_label),
    color = "black",
    linetype = "longdash",
    size = 0.8
  ) +
  geom_point(
    data = plot_df,
    aes(x = delta, y = model_label, fill = metric_label),
    shape = 21,
    color = "black",
    size = 7,
    stroke = 1.2
  ) +
  scale_x_continuous(
    limits = c(0, max_delta),
    breaks = seq(0, max_delta, by = 5),
    minor_breaks = seq(0, max_delta, by = 2.5),
    expand = c(0, 0)
  ) +
  scale_fill_manual(
```

```
    name = NULL,
    values = c("ΔBIC" = "black", "ΔLOOIC" = "white")
  ) +
  guides(
    fill = guide_legend(
      override.aes = list(shape = 21, size = 7, color = "black", stroke = 1.2),
      nrow = 1
    )
  ) +
  labs(
    x = paste0("Δ from Best Model (ΔBIC or ΔLOOIC)\n(best = ",
               combined_df$Model[which.min(na.omit(combined_df$Delta_BIC))], ")"),
    y = " M O D E L S"
  ) +
  theme_minimal(base_size = 20) +
  theme(
    legend.position = "top",
    legend.direction = "horizontal",
    legend.text = element_text(size = 20),
    legend.key.width = unit(0.5, "cm"),
    legend.spacing.x = unit(1.0, "cm"),
    plot.background = element_rect(fill = "white", color = NA),
    panel.background = element_rect(fill = "white", color = NA),
    axis.ticks.length = unit(0.3, "cm"),
    axis.ticks = element_line(size = 0.8),
    axis.ticks.x = element_line(color = "black"),
    axis.ticks.y = element_line(color = "black"),
    axis.text.x = element_text(size = 20),
    axis.text.y = element_text(size = 16, hjust = 0),
    axis.title.x = element_text(size = 20, face = "bold"),
    axis.title.y = element_text(size = 20, face = "bold", margin = margin(r = 10))
  )
#11 Define the specific output path for 400 DPI plot [note: the location of the saved figure is dependent on where the user has stored the file]

combined_plot_file_400 <- "C:/Users/gzavorsky/OneDrive - UC Davis Health/Current Working Manuscripts/COVID-19 paper/Figures/Figure_combined_BIC_LOOIC_400dpi.tiff"
combined_plot_dir <- dirname(combined_plot_file_400)

#12 Create directory if it doesn't exist
if (!dir.exists(combined_plot_dir)) {
  dir.create(combined_plot_dir, recursive = TRUE)
  cat("Created directory:", combined_plot_dir, "\n")
}
# Save 400 DPI plot
ggsave(
  filename = combined_plot_file_400,  plot = final_plot,  width = 19,  height = 13,
  dpi = 400,  compression = "lzw",  device = "tiff"
)
cat("✅ Combined plot (400 DPI) saved to:\n", combined_plot_file_400, "\n")
```

Model summaries and fit; Confusion-matrix counts (TP, TN, FP, FN) at Youden's J–optimal threshold, ranked by MCC

```
#———————————————————————————————————————
# 1. LOAD LIBRARIES & SET PATHS [note: the location of the saved results are dependent on where the user has stored the file(s)]
————————————————————————————————————————
library(lme4)
library(broom.mixed)
library(performance)
library(MuMIn)
library(pROC)
library(dplyr)
library(purrr)
library(tibble)
library(flextable)
library(officer)
library(haven)

data_path <- "C:/Users/gzavorsky/OneDrive - UC Davis Health/Desktop/Mendeley data Version 3 June 2025/Covid19_&_Controls_Filtered.sav"
save_path <- "C:/Users/gzavorsky/OneDrive - UC Davis Health/Current Working Manuscripts/COVID-19 paper/SPSS Files"

#———————————————————————————————————————
# 2. LOAD & PREPROCESS
#———————————————————————————————————————
covid_data <- read_sav(data_path) |>
  zap_labels() |>
  mutate(across(where(is.labelled), as.numeric))

# ——————————————————————————————————————
# 3. SPECIFY & FIT MODELS
# ——————————————————————————————————————
model_formulas <- list(
  "Two predictor model (DLNO\u2085\u209B, DLCO\u2085\u209B z-scores, GAMLSS)" =
    Disease ~ DLNO_Zscores_GAMLSS + DLCO_Zscores_GAMLSS + (1 | Study_Location),

  "Two predictor model (DLNO\u2085\u209B, DLCO\u2085\u209B z-scores, SLR)" =
    Disease ~ DLNO_Zscores_Segmented + DLCO_Zscores_Segmented + (1 | Study_Location),

  "Two predictor model (DLNO\u2085\u209B, DLCO\u2085\u209B z-scores, Munkholm et al. (2018))" =
    Disease ~ DLNO_Zscores_Munkholm + DLCO_Zscores_Munkholm + (1 | Study_Location),

  "Three predictor model (DLNO\u2085\u209B, DLCO\u2085\u209B, VA\u2085\u209B z-scores,
GAMLSS)" = Disease ~ DLNO_Zscores_GAMLSS + DLCO_Zscores_GAMLSS+VA_Zscores_GAMLSS
+ (1 | Study_Location),
```

"Three predictor model (DLNO\u2085\u209B, DLCO\u2085\u209B, VA\u2085\u209B z-scores, SLR)" = Disease ~ DLNO_Zscores_Segmented + DLCO_Zscores_Segmented + VA_Zscores_Segmented + (1 | Study_Location),

"Three predictor model (DLNO\u2085\u209B, DLCO\u2085\u209B, VA\u2085\u209B z-scores, Munkholm et al. (2018))" = Disease ~ DLNO_Zscores_Munkholm + DLCO_Zscores_Munkholm + VA_Zscores_Munkholm + (1 | Study_Location),

" TLC z-scores, GLI equations, Hall et al. (2021)" = Disease ~ TLC_Zscores + (1 | Study_Location),

"FEV\u2081/FVC z-scores, GLI equations, Quanjer et al. (2012)" = Disease ~ FEV1_FVC_Ratio_Zscores + (1 | Study_Location),

"FVC z-scores, GLI equations, Quanjer et al. (2012)" = Disease ~ FVC_Zscores + (1 | Study_Location),

"FEV\u2081 z-scores, GLI equations, Quanjer et al. (2012)" = Disease ~ FEV1_Zscores + (1 | Study_Location),

"VA-only z-scores, GLI equations, Stanojevic et al. (2017)" = Disease ~ VA_Zscores_GLI + (1 | Study_Location),

VA\u2085\u209B-only z-scores, SLR" = Disease ~ VA_Zscores_Segmented + (1 | Study_Location),

"VA\u2085\u209B-only z-scores, GAMLSS" = Disease ~ VA_Zscores_GAMLSS + (1 | Study_Location),

"VA\u2085\u209B-only z-scores, Munkholm et al. (2018)" = Disease ~ VA_Zscores_Munkholm + (1 | Study_Location), "DLCO-only z-scores, GLI equations, Stanojevic et al. (2017)" = Disease ~ DLCO_Zscores_GLI + (1 | Study_Location),

"DLCO\u2085\u209B-only z-scores, GAMLSS" = Disease ~ DLCO_Zscores_GAMLSS + (1 | Study_Location),

"DLCO\u2085\u209B-only z-scores, SLR" = Disease ~ DLCO_Zscores_Segmented + (1 | Study_Location),

"DLCO\u2085\u209B-only z-scores, Munkholm et al. (2018)" = Disease ~ DLCO_Zscores_Munkholm + (1 | Study_Location),

"DLNO\u2085\u209B-only z-scores, GAMLSS" = Disease ~ DLNO_Zscores_GAMLSS + (1 | Study_Location),

"DLNO\u2085\u209B-only z-scores, SLR" = Disease ~ DLNO_Zscores_Segmented + (1 | Study_Location),

"DLNO\u2085\u209B-only z-scores, Munkholm et al. (2018)" =Disease ~ DLNO_Zscores_Munkholm + (1 | Study_Location),

"Summed DLNO\u2085\u209B+DLCO\u2085\u209B z-scores, GAMLSS" = Disease ~ Combined_Zscores_GAMLSS + (1 | Study_Location),

```
"Summed DLNO\u2085\u209B+DLCO\u2085\u209B z-scores, SLR" = Disease ~
Combined_Zscores_Segmented + (1 | Study_Location),

"Summed DLNO\u2085\u209B+DLCO\u2085\u209B z-scores, Munkholm et al. (2018)" =
   Disease ~ Combined_Zscores_Munkholm + (1 | Study_Location))

fitted_models <- map(model_formulas, ~ glmer(.x, data = covid_data, family = binomial, control =
glmerControl(optimizer = "bobyqa")))

# ──────────────────────────────────────────────────────────────
# 4. SUPPORTING FUNCTIONS
# ──────────────────────────────────────────────────────────────

# 4a. Model summaries & fit (table_model_summaries)
extract_summary <- function(model, model_name) {
  summ    <- broom.mixed::tidy(model, effects = "fixed", conf.int = TRUE)
  r2_vals <- MuMIn::r.squaredGLMM(model)["delta", ]
  pct     <- if (r2_vals[2] > 0) round(100 * r2_vals[1] / r2_vals[2], 0) else NA_real_

  int <- summ |>
    filter(term == "(Intercept)") |>
    transmute(
      Model               = model_name,
      Predictor           = "Intercept",
      "Estimate (95% CI)" = sprintf("%.2f [%.2f, %.2f]", estimate, conf.low, conf.high),
      SE                  = sprintf("%.2f", std.error),
      "p-value"           = ifelse(p.value < 0.0001, "<0.0001", sprintf("%.3f", p.value)),
      "OR (95% CI)"       = "",
      AIC                 = sprintf("%d", round(AIC(model))),
      BIC                 = sprintf("%d", round(BIC(model))),
      "Marginal R²"       = sprintf("%.2f", r2_vals[1]),
      "Random Effect R²"  = sprintf("%.2f", r2_vals[2] - r2_vals[1]),
      "Variance Explained %" = sprintf("%d", pct),
      Row_Order           = 1
    )

  sd_site <- as.data.frame(VarCorr(model)) |>
    filter(grp == "Study_Location", var1 == "(Intercept)") |>
    pull(sdcor)

  rand <- tibble(
    Model               = "",
    Predictor           = "SD of random intercept: Study Site",
    "Estimate (95% CI)" = sprintf("%.2f", sd_site),
    SE                  = "",
    "p-value"           = NA_character_,
    "OR (95% CI)"       = NA_character_,
    AIC                 = "",
    BIC                 = "",
    "Marginal R²"       = "",
    "Random Effect R²"  = "",
```

```
  "Variance Explained %" = "",
  Row_Order         = 3
 )

 coefs <- summ |>
  filter(term != "(Intercept)") |>
  transmute(
   Model            = "",
   Predictor         = recode(
    term,
    DLNO_Zscores_Segmented     = "DLNO\u2085\u209B z-scores, SLR",
    DLCO_Zscores_Segmented     = "DLCO\u2085\u209B z-scores, SLR",
    VA_Zscores_Segmented       = "VA\u2085\u209B z-scores, SLR",
    DLNO_Zscores_GAMLSS        = "DLNO\u2085\u209B z-scores, GAMLSS",
    DLCO_Zscores_GAMLSS        = "DLCO\u2085\u209B z-scores, GAMLSS",
    VA_Zscores_GAMLSS          = "VA\u2085\u209B z-scores, GAMLSS",
     Combined_Zscores_GAMLSS    = "Summed DLNO\u2085\u209B+DLCO\u2085\u209B,
    GAMLSS",
     Combined_Zscores_Munkholm  = "Summed DLNO\u2085\u209B+DLCO\u2085\u209B,
    Munkholm et al. (2018)",
    DLNO_Zscores_Munkholm      = "DLNO\u2085\u209B z-scores, Munkholm et al. (2018)",
    DLCO_Zscores_Munkholm      = "DLCO\u2085\u209B z-scores, Munkholm et al. (2018)",
    VA_Zscores_Munkholm        = "VA\u2085\u209B z-scores, Munkholm et al. (2018)",
    DLCO_Zscores_GLI           = "DLCO z-scores, GLI",
    VA_Zscores_GLI             = "VA z-scores, GLI",
    FVC_Zscores                = "FVC z-scores, GLI",
    FEV1_Zscores               = "FEV\u2081 z-scores, GLI",
    FEV1_FVC_Ratio_Zscores     = "FEV\u2081/FVC z-scores, GLI",
    TLC_Zscores                = "TLC z-scores, GLI",
    .default                   = term
   ),
   "Estimate (95% CI)" = sprintf("%.2f [%.2f, %.2f]", estimate, conf.low, conf.high),
   SE                = sprintf("%.2f", std.error),
   "p-value"          = ifelse(p.value < 0.0001, "<0.0001", sprintf("%.3f", p.value)),
   "OR (95% CI)"       = sprintf("%.2f [%.2f, %.2f]", exp(estimate), exp(conf.low), exp(conf.high)),
   AIC               = "",
   BIC               = "",
   "Marginal R²"       = "",
   "Random Effect R²"  = "",
   "Variance Explained %" = "",
   Row_Order          = 2
  )  bind_rows(int, coefs, rand) |>
  mutate(Model_Group = model_name)}
```

# 4b. Threshold-based metrics & z-scores (table_threshold)

```
get_threshold_with_z <- function(model, model_name) {
 pred    <- predict(model, type = "response")
 actual  <- model@frame$Disease
 roc_obj <- suppressMessages(pROC::roc(actual, pred, direction = "<", quiet = TRUE))
 cb      <- coords(roc_obj, "best", best.method = "youden")
 thr     <- as.numeric(cb["threshold"])
```

```r
  youden  <- as.numeric(cb["sensitivity"]) + as.numeric(cb["specificity"]) - 1
  logit   <- log(thr / (1 - thr))

  # Define expected z-scores based on model name
  expected_z_scores <- c(
    "Three predictor model (DLNO5s, DLCO5s, VA5s z-scores, SLR)" = NA,
    "Three predictor model (DLNO5s, DLCO5s, VA5s z-scores, GAMLSS)" = NA,
    "Two predictor model (DLNO5s, DLCO5s z-scores, GAMLSS)" = NA,
    "Summed DLNO5s+DLCO5s z-scores, GAMLSS" = -4.12,
    "Two predictor model (DLNO5s, DLCO5s z-scores, SLR)" = NA,
    "Two predictor model (DLNO5s, DLCO5s z-scores, Munkholm et al. (2018))" = NA,
    "Summed DLNO5s+DLCO5s z-scores, SLR" = -2.69,
    "Summed DLNO5s+DLCO5s z-scores, Munkholm et al. (2018)" = -2.91,
    "DLNO5s-only z-scores, Munkholm et al. (2018)" = -2.01,
    "DLNO5s-only z-scores, GAMLSS" = -2.47,
    "Three predictor model (DLNO5s, DLCO5s, VA5s z-scores, Munkholm et al. (2018))" = NA,
    "DLCO5s-only z-scores, GAMLSS" = -1.69,
    "DLNO5s-only z-scores, SLR" = -2.05,
    "DLCO-only z-scores, GLI equations, Stanojevic et al. (2017)" = -0.68,
    "DLCO5s-only z-scores, SLR" = -1.37,
    "DLCO5s-only z-scores, Munkholm et al. (2018)" = -2.02,
    "VA5s-only z-scores, SLR" = -1.21,
    "TLC z-scores, GLI equations, Hall et al. (2021)" = -0.05,
    "VA5s-only z-scores, GAMLSS" = -0.81,
    "VA-only z-scores, GLI equations, Stanojevic et al. (2017)" = -0.81,
    "VA5s-only z-scores, Munkholm et al. (2018)" = -1.00,
    "FVC z-scores, GLI equations, Quanjer et al. (2012)" = -1.52,
    "FEV1 z-scores, GLI equations, Quanjer et al. (2012)" = -1.16,
    "FEV1/FVC z-scores, GLI equations, Quanjer et al. (2012)" = 0.88
  )

  cz <- expected_z_scores[[model_name]]  # Use pre-defined expected z-scores

  tibble(
    Model                  = model_name,
    Threshold              = round(thr, 3),
    "Youden's J"           = round(youden, 3),
    "Youden Z-Score"       = round(logit, 3),
    "Closest predictor z-score" = round(cz, 3)
  )
}

table_threshold <- map2_dfr(fitted_models, names(fitted_models), get_threshold_with_z) |>
  slice(match(table_classification_performance_metrics$Model, Model))
print(table_threshold)

# 4c. Classification performance metrics (table_classification_performance_metrics)
get_metrics <- function(model, model_name) {
  pred      <- predict(model, type = "response")
  actual    <- model@frame$Disease
```

```
  roc_obj    <- suppressMessages(pROC::roc(actual, pred, direction = "<", quiet = TRUE))
  thr        <- coords(roc_obj, "best", ret = "threshold", best.method = "youden")$threshold
  class_pred <- ifelse(pred > thr, 1, 0)
  cm         <- table(
    factor(class_pred, levels = c(0, 1)),
    factor(actual,     levels = c(0, 1))
  )

  tn <- as.numeric(cm[1,1])
  fp <- as.numeric(cm[2,1])
  fn <- as.numeric(cm[1,2])
  tp <- as.numeric(cm[2,2])

  sens    <- tp / (tp + fn)
  spec    <- tn / (tn + fp)
  fpr     <- fp / (fp + tn)
  fnr     <- fn / (fn + tp)
  ppv     <- tp / (tp + fp)
  npv     <- tn / (tn + fn)
  fdr     <- fp / (tp + fp)
  for_    <- fn / (tn + fn)

  auc_ci  <- ci.auc(roc_obj)
  mcc_num <- tp * tn - fp * fn
  mcc_den <- sqrt((tp + fp) * (tp + fn) * (tn + fp) * (tn + fn))
  mcc     <- if (mcc_den > 0) mcc_num / mcc_den else NA_real_

  tibble(
    Model              = model_name,
    Misclassified      = fp + fn,
    "% Misclassified"  = round(100 * (fp + fn) / sum(cm), 1),
    "AUROC (95% CI)"   = sprintf("%.2f [%.2f, %.2f]", auc_ci[2], auc_ci[1], auc_ci[3]),
    MCC                = round(mcc, 3),
    Sensitivity        = round(sens, 2),
    Specificity        = round(spec, 2),
    "Balanced Accuracy" = round((sens + spec) / 2, 2),
    FPR                = round(fpr, 2),
    FNR                = round(fnr, 2),
    FDR                = round(fdr, 2),
    FOR                = round(for_, 2),
    PPV                = round(ppv, 2),
    NPV                = round(npv, 2)
  )
}

# 4d. Confusion-matrix counts (table_confusion_matrix_counts)
get_confusion_counts <- function(model, model_name) {
  pred       <- predict(model, type = "response")
  actual     <- model@frame$Disease
  roc_obj    <- suppressMessages(pROC::roc(actual, pred, direction = "<", quiet = TRUE))
  thr        <- coords(roc_obj, "best", ret = "threshold", best.method = "youden")$threshold
```

```
  class_pred <- ifelse(pred > thr, 1, 0)
  cm         <- table(
    factor(class_pred, levels = c(0, 1)),
    factor(actual,     levels = c(0, 1))
  )

  tn <- as.numeric(cm[1,1])
  fp <- as.numeric(cm[2,1])
  fn <- as.numeric(cm[1,2])
  tp <- as.numeric(cm[2,2])

  mcc_num <- tp * tn - fp * fn
  mcc_den <- sqrt((tp + fp) * (tp + fn) * (tn + fp) * (tn + fn))
  mcc     <- if (mcc_den > 0) mcc_num / mcc_den else NA_real_

  tibble(
    Model = model_name,
    TP    = tp,
    TN    = tn,
    FP    = fp,
    FN    = fn,
    MCC   = round(mcc, 3)
  )
}

#———————————————————————————————————————————
# 5. BUILD TABLES
#———————————————————————————————————————————
table_model_summaries <- map2_dfr(fitted_models, names(fitted_models), extract_summary)

# Sort table_model_summaries (lowest to highest), preserving model group order
model_bics <- table_model_summaries |>
  filter(Model != "") |>
  select(Model, BIC, Model_Group) |>
  mutate(BIC = as.numeric(BIC)) |>
  distinct()

model_order <- model_bics |>
  arrange(BIC) |>
  pull(Model_Group)

table_model_summaries <- table_model_summaries |>
  mutate(Model_Group = factor(Model_Group, levels = model_order)) |>
  arrange(Model_Group, Row_Order) |>
  select(-Model_Group, -Row_Order) |>
  mutate(Model = as.character(Model))

table_classification_performance_metrics <- map2_dfr(fitted_models, names(fitted_models),
get_metrics) |>
  arrange(desc(MCC))
```

```
table_confusion_matrix_counts <- map2_dfr(fitted_models, names(fitted_models),
get_confusion_counts) |>
  slice(match(table_classification_performance_metrics$Model, Model))

table_threshold <- map2_dfr(fitted_models, names(fitted_models), get_threshold_with_z) |>
  slice(match(table_classification_performance_metrics$Model, Model))

#------------------------------------------------------------------------------
# 6. EXPORT TO WORD
#------------------------------------------------------------------------------

# Table Model summaries (landscape): Model summaries and fit
ft_table_model_summaries <- flextable(table_model_summaries) |>
  autofit() |>
  fontsize(size = 9, part = "all") |>
  merge_v(j = "Model") |>
  align(j = "Predictor", align = "left", part = "all") |>
  align(j = c("Estimate (95% CI)", "SE", "OR (95% CI)"), align = "center", part = "all") |>
  align(j = setdiff(names(table_model_summaries), c("Model", "Predictor", "Estimate (95% CI)", "SE",
"OR (95% CI)")), align = "center", part = "all") |>
  width(j = "Estimate (95% CI)", width = 2.0) |>
  width(j = "SE", width = 0.8) |>
  width(j = "OR (95% CI)", width = 2.0) |>
  set_table_properties(layout = "autofit", width = 1)

doc_table_model_summaries <- read_docx() |>
  body_add_par("Table. Model summaries and fit.", style = "heading 1") |>
  body_add_flextable(ft_table_model_summaries) |>

print(doc_table_model_summaries, target = file.path(save_path,
"table_model_summaries.docx"))

#6a table_threshold (portrait): Threshold-based metrics and associated z-scores
ft_table_threshold <- flextable(table_threshold) |>
  autofit() |>
  fontsize(size = 9, part = "all") |>
  align(part = "all", align = "center") |>
  set_table_properties(layout = "autofit", width = 1)

doc_table_threshold <- read_docx() |>
  body_add_par(
    "Threshold-based metrics and associated z-scores for model discrimination at a probability threshold
that maximizes Youden’s J, ranked by MCC.",
    style = "heading 1"
  ) |>
  body_add_flextable(ft_table_threshold) |>
  body_end_section_portrait()

print(doc_table_threshold, target = file.path(save_path, "table_threshold.docx"))}
```

```
# table_confusion_matrix_counts (portrait): Confusion-matrix counts

ft_table_confusion_matrix_counts <- flextable(table_confusion_matrix_counts) |>
  autofit() |>
  fontsize(size = 9, part = "all") |>
  align(part = "all", align = "center") |>
  set_table_properties(layout = "autofit", width = 1)

doc_table_confusion_matrix_counts <- read_docx() |>
  body_add_par(
    "Confusion-matrix counts (TP, TN, FP, FN) at Youden’s J–optimal threshold, ranked by MCC (n =
644).",
    style = "heading 1"
  ) |>
  body_add_flextable(ft_table_confusion_matrix_counts) |>
  body_end_section_portrait()

print(doc_table_confusion_matrix_counts, target = file.path(save_path,
"table_confusion_matrix_counts.docx"))

# table_classification_performance_metrics (landscape): Classification performance metrics

ft_table_classification_performance_metrics <- flextable(table_classification_performance_metrics) |>
  autofit() |>
  fontsize(size = 9, part = "all") |>
  align(part = "all", align = "center") |>
  width(j = "AUROC (95% CI)", width = 2.2) |>
  set_table_properties(layout = "autofit", width = 1)

doc_table_classification_performance_metrics <- read_docx() |>
  body_add_par(
    "Classification performance metrics ranked by MCC. The Threshold selection was chosen to
maximize Youden’s J on each model’s ROC.",
    style = "heading 1"
  ) |>
  body_add_flextable(ft_table_classification_performance_metrics) |>
  body_end_section_landscape()

print(doc_table_classification_performance_metrics, target = file.path(save_path,
"table_classification_performance_metrics.docx"))
```

Model performance summary when adding $DLNO_{5s}$ z-scores to the best $DLCO_{5s}$-only z-scores model.

```
# ─────────────────────────────────────────────
# 1. LOAD LIBRARIES & SET PATHS [note: the location of the data is dependent on where the user has stored the file]. As well, saving it to a specific location is dependent on the user]

# ─────────────────────────────────────────────
library(lme4)
library(broom.mixed)
library(performance)
library(MuMIn)
library(pROC)
library(dplyr)
library(purrr)
library(tibble)
library(flextable)
library(officer)
library(haven)
library(boot)
library(nricens)

data_path <- "C:/Users/gzavorsky/OneDrive - UC Davis Health/Desktop/Mendeley data Version 3 June 2025/Covid19_&_Controls_Filtered.sav"
save_path <- "C:/Users/gzavorsky/OneDrive - UC Davis Health/Current Working Manuscripts/COVID-19 paper/SPSS Files"

# ─────────────────────────────────────────────
# 2. LOAD & PREPROCESS
# ─────────────────────────────────────────────
covid_data <- read_sav(data_path) |>
  zap_labels() |>
  mutate(across(where(is.labelled), as.numeric))

# ─────────────────────────────────────────────
# 3. FIT GLMM MODELS (DLCO-only vs Combined DLCO+DLNO)
# ─────────────────────────────────────────────
covid_data <- covid_data |>
  mutate(
    disease = factor(Disease, levels = c(0, 1))
  ) |>
  select(-Disease)

model_dlco <- glmer(
  disease ~ DLCO_Zscores_GAMLSS + (1 | Study_Location),
  data = covid_data,
  family = binomial
)
```

```r
model_combined <- glmer(
  disease ~ Combined_Zscores_GAMLSS + (1 | Study_Location),
  data = covid_data,
  family = binomial
)

# ──────────────────────────────────────────────────────────────
# 4. PREDICT & ROC CURVES
# ──────────────────────────────────────────────────────────────
prob_dlco     <- predict(model_dlco,     type = "response")
prob_combined <- predict(model_combined, type = "response")

roc_dlco     <- roc(covid_data$disease, prob_dlco)
roc_combined <- roc(covid_data$disease, prob_combined)

# ──────────────────────────────────────────────────────────────
# 5. FUNCTION TO EXTRACT YOUDEN'S J & CI
# ──────────────────────────────────────────────────────────────
extract_youden <- function(roc_obj, boot_n = 10000) {
  coords_best <- coords(roc_obj, x = "best", best.method = "youden", transpose = FALSE)
  ci_list <- ci.coords(roc_obj, x = "best", best.method = "youden", ret = "youden", boot.n = boot_n)

  youden_val <- as.numeric(coords_best$youden) - 1
  ci_vals    <- as.numeric(unlist(ci_list)) - 1

  list(
    youden = youden_val,
    ci     = c(lower = ci_vals[1], mid = ci_vals[2], upper = ci_vals[3])
  )
}

res_dlco     <- extract_youden(roc_dlco)
res_combined <- extract_youden(roc_combined)

# ──────────────────────────────────────────────────────────────
# 6. CALCULATE NRI
# ──────────────────────────────────────────────────────────────
nri_result <- nribin(
  event  = covid_data$disease,
  p.std  = prob_dlco,
  p.new  = prob_combined,
  updown = "diff",
  cut    = 0,
  niter  = 10000
)

# ──────────────────────────────────────────────────────────────
# 7. BOOTSTRAP IDI
# ──────────────────────────────────────────────────────────────
idi_stat <- function(data, indices) {
  d  <- data[indices, ]
```

```
  p0 <- predict(model_dlco,     newdata = d, type = "response")
  p1 <- predict(model_combined, newdata = d, type = "response")

  (mean(p1[d$disease == 1]) - mean(p0[d$disease == 1])) -
    (mean(p1[d$disease == 0]) - mean(p0[d$disease == 0]))
}

set.seed(2025)
idi_boot <- boot(
  data      = covid_data,
  statistic = idi_stat,
  R         = 10000,
  parallel  = "multicore",
  ncpus     = parallel::detectCores()
)

idi_ci <- boot.ci(idi_boot, type = "perc")

# ───────────────────────────────────────────────
# 8. BUILD TABLE
# ───────────────────────────────────────────────
youden_dlco_tbl <- tibble(
  Metric    = "Youden J (DLCO)",
  Estimate  = round(res_dlco$youden, 3),
  Std.Error = NA_real_,
  Lower     = round(res_dlco$ci["lower"], 3),
  Upper     = round(res_dlco$ci["upper"], 3))

youden_comb_tbl <- tibble(
  Metric    = "Youden J (Combined)",
  Estimate  = round(res_combined$youden, 3),
  Std.Error = NA_real_,
  Lower     = round(res_combined$ci["lower"], 3),
  Upper     = round(res_combined$ci["upper"], 3))

nri_tbl <- as.data.frame(nri_result$nri) |>
  rownames_to_column("Metric") |>
  select(Metric, Estimate, Std.Error, Lower, Upper) |>
  mutate(across(c(Estimate, Std.Error, Lower, Upper), round, 3))

idi_tbl <- tibble(
  Metric    = "IDI",
  Estimate  = round(idi_boot$t0, 3),
  Std.Error = NA_real_,
  Lower     = round(idi_ci$percent[4], 3),
  Upper     = round(idi_ci$percent[5], 3))

summary_df <- bind_rows(youden_dlco_tbl, youden_comb_tbl, nri_tbl, idi_tbl)
```

```
# ---------------------------------------------------------------------------
# 9. EXPORT WORD DOCUMENT [note: the location of where the word document is saved dependent on where the user has saved the file]
# ---------------------------------------------------------------------------
ft_table <- flextable(summary_df) |>
  set_header_labels(
    Metric    = "Metric",
    Estimate  = "Estimate",
    Std.Error = "Std. Error",
    Lower     = "95% CI Lower",
    Upper     = "95% CI Upper"
  ) |>
  autofit()

doc <- read_docx() |>
  body_add_par(
    "Model performance summary when adding DLNO5s z-scores to the best DLCO5s-only z-scores model.",
    style = "heading 2"
  ) |>
  body_add_flextable(ft_table)

print(doc, target = file.path(save_path, "Table_Model_Performance_summary.docx"))
```

Agreement Analysis

```
#———————————————————————————————————————————————
# 0.   PACKAGES
#———————————————————————————————————————————————
library(haven)       # for read_sav()
library(labelled)    # for zap_labels()
library(dplyr)       # data-wrangling
library(irr)         # kappa2()
library(purrr)       # map_*
library(tidyr)       # pivoting if needed
library(readr)       # write_csv()
library(officer)     # Word output
library(flextable)   # for Word tables

#———————————————————————————————————————————————
#1.   PATHS & LOAD DATA [note: the location of the data is dependent on where the user has stored the file. As such, this step will be dependent on where the user saved the file Covid19_&_Controls_Filtered.sav]
#———————————————————————————————————————————————

data_path <- "C:/Users/gzavorsky/OneDrive - UC Davis Health/Desktop/Mendeley data Version 3 June 2025/Covid19_&_Controls_Filtered.sav"
save_path <- "C:/Users/gzavorsky/OneDrive - UC Davis Health/Current Working Manuscripts/COVID-19 paper"

dir.create(save_path, recursive = TRUE, showWarnings = FALSE)

covid_data <-
  read_sav(data_path) %>%
  zap_labels() %>%
  mutate(across(where(is.labelled), as.numeric)) %>%
  # create below-LLN flags:
  mutate(
    below_dlco_gamlss = DLCO_Zscores_GAMLSS < -1.645,
    below_dlno_gamlss = DLNO_Zscores_GAMLSS < -1.645,
    below_dlco_slr    = DLCO_Zscores_Segmented < -1.645,
    below_dlno_slr    = DLNO_Zscores_Segmented < -1.645,
    below_dlco_munk   = DLCO_Zscores_Munkholm < -1.645,
    below_dlno_munk   = DLNO_Zscores_Munkholm < -1.645
  )

#———————————————————————————————————————————————
#2.   UTILITY FUNCTIONS
#———————————————————————————————————————————————
interpret_kappa <- function(kappa) {
  dplyr::case_when(
    kappa <= 0.20 ~ "None",
    kappa <= 0.39 ~ "Minimal",
    kappa <= 0.59 ~ "Weak",
```

```
    kappa <= 0.79 ~ "Moderate",
    kappa <= 0.90 ~ "Strong",
    kappa >  0.90 ~ "Almost Perfect",
    TRUE          ~ NA_character_
  )
}
compute_metrics <- function(data, col_dlco, col_dlno,
                            n_boot = 10000, seed = 123) {
  dlco_flag <- data[[col_dlco]]
  dlno_flag <- data[[col_dlno]]

  # point estimates
  κ_obs <- irr::kappa2(
    data.frame(dlco_flag, dlno_flag)
  )$value

  missed_dlco_obs <- sum(!dlco_flag & dlno_flag) /
    sum(dlno_flag) * 100
  missed_dlno_obs <- sum(!dlno_flag & dlco_flag) /
    sum(dlco_flag) * 100

  # bootstrap
  set.seed(seed)
  boots <- replicate(
    n_boot,
    {
      idx   <- sample.int(nrow(data), replace = TRUE)
      d_b   <- dlco_flag[idx]
      n_b   <- dlno_flag[idx]
      κ_b   <- irr::kappa2(data.frame(d_b, n_b))$value
      mCO_b <- sum(!d_b & n_b) / sum(n_b) * 100
      mNO_b <- sum(!n_b & d_b) / sum(d_b) * 100
      c(kapp = κ_b, missCO = mCO_b, missNO = mNO_b)
    },
    simplify = "matrix"
  )
  cis <- apply(boots, 1, quantile, probs = c(0.025, 0.975), na.rm = TRUE)

  tibble::tibble(
    kappa           = κ_obs,
    kappa_lower      = cis["2.5%", "kapp"],
    kappa_upper      = cis["97.5%", "kapp"],
    missed_dlco      = missed_dlco_obs,
    missed_dlco_low  = cis["2.5%", "missCO"],
    missed_dlco_high = cis["97.5%", "missCO"],
    missed_dlno      = missed_dlno_obs,
    missed_dlno_low  = cis["2.5%", "missNO"],
    missed_dlno_high = cis["97.5%", "missNO"]
  )
}
```

```
#————————————————————————————————————————————————
# 3.   RUN ANALYSES
#————————————————————————————————————————————————
results_gamlss <- compute_metrics(
  covid_data, "below_dlco_gamlss", "below_dlno_gamlss"
)

results_slr <- compute_metrics(
  covid_data, "below_dlco_slr",    "below_dlno_slr"
)

results_munk <- compute_metrics(
  covid_data, "below_dlco_munk",   "below_dlno_munk"
)

final_table <-
  dplyr::bind_rows(
    results_gamlss %>% dplyr::mutate(reference = "GAMLSS"),
    results_slr    %>% dplyr::mutate(reference = "SLR"),
    results_munk   %>% dplyr::mutate(reference = "Munkholm et al. (2018)")
  ) %>%
  dplyr::mutate(
    kappa_ci       = sprintf("%.2f (%.2f–%.2f)",
                         kappa, kappa_lower, kappa_upper),
    missed_dlco_ci = sprintf("%.1f%% (%.1f–%.1f)",
                         missed_dlco, missed_dlco_low, missed_dlco_high),
    missed_dlno_ci = sprintf("%.1f%% (%.1f–%.1f)",
                         missed_dlno, missed_dlno_low, missed_dlno_high),
    Interpretation = interpret_kappa(kappa),
    winner         = dplyr::if_else(
      missed_dlco < missed_dlno, "DLCO",
      dplyr::if_else(missed_dlno < missed_dlco, "DLNO", "Tie")
    )
  ) %>%
  dplyr::select(
    `Reference equations used`      = reference,
    `Kappa (95% CI)`                = kappa_ci,
    Interpretation,
    `Missed (%) if only DLCO used`  = missed_dlco_ci,
    `Missed (%) if only DLNO used`  = missed_dlno_ci,
    `Overall winner`     = winner)

# — or for a prettier ASCII-table in the console —
knitr::kable(final_table)

#————————————————————————————————————————————————
# 4.   TEST SIGNIFICANT DIFFERENCE IN MISSED-CASE %
#————————————————————————————————————————————————

# 4.1 Bootstrap the difference in missed-case percentages ---------------------------------------------------
bootstrap_diff <- function(data, col_dlco, col_dlno,
```

```
                         n_boot = 10000, seed = 123) {
  # extract flags
  dlco_flag <- data[[col_dlco]]
  dlno_flag <- data[[col_dlno]]

  # observed missed-case percentages
  missed_dlco_obs <- sum(!dlco_flag & dlno_flag) / sum(dlno_flag) * 100
  missed_dlno_obs <- sum(!dlno_flag & dlco_flag) / sum(dlco_flag) * 100
  diff_obs <- missed_dlco_obs - missed_dlno_obs

  # bootstrap replicates of the difference
  set.seed(seed)
  diffs <- replicate(
    n_boot,
    {
      idx <- sample.int(nrow(data), replace = TRUE)
      d_b <- dlco_flag[idx]
      n_b <- dlno_flag[idx]
      mco <- sum(!d_b & n_b) / sum(n_b) * 100
      mno <- sum(!n_b & d_b) / sum(d_b) * 100
      mco - mno
    }
  )
  ci   <- quantile(diffs, probs = c(0.025, 0.975), na.rm = TRUE)
  pval <- 2 * min(mean(diffs >= 0), mean(diffs <= 0))

  tibble::tibble(
    diff_obs   = diff_obs,    diff_lower = ci[[1]],    diff_upper = ci[[2]],    p_value    = pval )
}
#4.2 apply to each reference equation
for (ref in c("gamlss", "slr", "munk")) {
  cat("\n=== Reference:", toupper(ref), "===\n")
  res_boot <- bootstrap_diff(
    covid_data,
    col_dlco = paste0("below_dlco_",  ref),
    col_dlno = paste0("below_dlno_",  ref)
  )
  print(res_boot)
}
# 4.3 McNemar's test on the paired below-LLN flags
# This tests H0: P(below_LL.) by DLCO == P(below_LL.) by DLNO
# i.e. is there a systematic difference in how often each method
# flags someone < LLN?
for (ref in c("gamlss", "slr", "munk")) {
  dlco_flag <- covid_data[[paste0("below_dlco_", ref)]]
  dlno_flag <- covid_data[[paste0("below_dlno_", ref)]]
  tab       <- table(dlco_flag, dlno_flag)
  cat("\n--- McNemar test, reference:", toupper(ref), "---\n")
  print(mcnemar.test(tab, correct = FALSE)) }
```

The Area Under the Receiver Operating Characteristic Curve (AUROC) and Mathews Correlation Coefficient (MCC)

```
#------------------------------------------------------------------------------------------------------------------------
#AUROC
#------------------------------------------------------------------------------------------------------------------------

#── 1) load all libraries
library(dplyr)
library(ggplot2)
library(blme)
library(pROC)
library(purrr)
library(haven)
library(performance)
library(lmtest)
library(lme4)
library(glmnet)
library(boot)
library(openxlsx)
library(tictoc)

#── 2) set seed & start timer
set.seed(123)
tic("Model evaluation time")

#── 3) prepare data (strip labelled class BEFORE modeling)
covid <- Covid19_Controls_Filtered %>%
  zap_labels() %>%                  # drop haven_labelled, keep raw 0/1 etc.
  filter(Machine == 0) %>%          # only Machine == 0
  slice_head(n = 644) %>%           # first 644 rows
  mutate(study = as_factor(Study))  # keep Study as a factor

#------------------------------------------------------------------------------------------------------------------------
#── 4) fit all candidate GLMMs
#------------------------------------------------------------------------------------------------------------------------
cand_models <- list(

# Spirometry and total lung capacity (TLC) z-scores models
  m_tlc      = glmer(Disease ~ TLC_Zscores  + (1 | Study_Location),
                  data = covid, family = binomial, control = glmerControl(optimizer = "bobyqa",
                                   optCtrl  = list(maxfun = 2e5), tolPwrss = 1e-4), nAGQ = 10),

  m_ratio    = glmer(Disease ~ FEV1_FVC_Ratio_Zscores + (1 | Study_Location),
                  data = covid, family = binomial, control = glmerControl(optimizer = "bobyqa",
                                   optCtrl  = list(maxfun = 2e6), tolPwrss = 1e-4),nAGQ = 10),
```

```
m_fvc      = glmer(Disease ~ FVC_Zscores  + (1 | Study_Location), data = covid, family = binomial,
               control = glmerControl(optimizer = "bobyqa", optCtrl  = list(maxfun = 2e5), tolPwrss = 1e-
              4), nAGQ = 10),


m_fev1     = glmer(Disease ~ FEV1_Zscores + (1 | Study_Location), data = covid, family = binomial,
               control = glmerControl(optimizer = "bobyqa", optCtrl  = list(maxfun = 2e5), tolPwrss = 1e-
                4), nAGQ = 10),

# Alveolar volume (VA) z-scores models
  m_va_gli       = glmer(Disease ~ VA_Zscores_GLI  + (1 | Study_Location), data = covid, family =
binomial, control = glmerControl(optimizer = "bobyqa", optCtrl  = list(maxfun = 2e6), tolPwrss = 1e-4),
                  nAGQ = 10),

  m_va_segmented  = glmer(Disease ~ VA_Zscores_Segmented + (1 | Study_Location),data = covid,
                  family = binomial, control = glmerControl(optimizer = "bobyqa",  optCtrl  = list(maxfun =
                  2e6),tolPwrss = 1e-4), nAGQ = 10),

m_va_gamlss     = glmer(Disease ~ VA_Zscores_GAMLSS   + (1 | Study_Location), data = covid,
                  family = binomial, control = glmerControl(optimizer = "bobyqa", optCtrl  = list(maxfun =
                  2e6), tolPwrss = 1e-4), nAGQ = 10),

  m_va_munkholm   = glmer(Disease ~ VA_Zscores_Munkholm  + (1 | Study_Location), data = covid,
                  family = binomial, control = glmerControl(optimizer = "bobyqa", optCtrl  = list(maxfun =
                  2e6), tolPwrss = 1e-4), nAGQ = 10),

  # pulmonary diffusing capacity for CO (DLCO) z-scores models
  m_dlco_gli       = glmer(Disease ~ DLCO_Zscores_GLI   + (1 | Study_Location), data = covid, family =
                  binomial, control = glmerControl(optimizer = "bobyqa", optCtrl  = list(maxfun = 2e6),
                  tolPwrss = 1e-4),nAGQ = 10),

  m_dlco_segmented = glmer(Disease ~ DLCO_Zscores_Segmented + (1 | Study_Location),
                  data = covid, family = binomial, control = glmerControl(optimizer = "bobyqa", optCtrl  =
                  list(maxfun = 2e6), tolPwrss = 1e-4), nAGQ = 10),


  m_dlco_gamlss    = glmer(Disease ~ DLCO_Zscores_GAMLSS    + (1 | Study_Location), data =
                   covid, family = binomial, control = glmerControl(optimizer = "bobyqa", optCtrl  =
                   list(maxfun = 2e6), tolPwrss = 1e-4), nAGQ = 10),

  m_dlco_munkholm  = glmer(Disease ~ DLCO_Zscores_Munkholm  + (1 | Study_Location), data =
                   covid, family = binomial, control = glmerControl(optimizer = "bobyqa", optCtrl  =
                   list(maxfun = 2e6), tolPwrss = 1e-4), nAGQ = 10),

  # pulmonary diffusing capacity for nitric oxide (DLNO) z-score models
  m_dlno_segmented = glmer(Disease ~ DLNO_Zscores_Segmented + (1 | Study_Location), data =
                    covid, family = binomial, control = glmerControl(optimizer = "bobyqa", optCtrl  =
                    list(maxfun = 2e6), tolPwrss = 1e-4),nAGQ = 10),
```

```
  m_dlno_gamlss    = glmer(Disease ~ DLNO_Zscores_GAMLSS    + (1 | Study_Location), data =
                           covid, family = binomial, control = glmerControl(optimizer = "bobyqa", optCtrl  =
                           list(maxfun = 2e6), tolPwrss = 1e-4), nAGQ = 10),

  m_dlno_munkholm  = glmer(Disease ~ DLNO_Zscores_Munkholm  + (1 | Study_Location), data =
                           covid, family = binomial, control = glmerControl(optimizer = "bobyqa", optCtrl  =
                           list(maxfun = 2e6), tolPwrss = 1e-4), nAGQ = 10),

# Summed “Combined” DLNO+DLCO z-scores models
  m_combined_segmented = glmer(Disease ~ Combined_Zscores_Segmented + (1 | Study_Location),
                           data = covid, family = binomial, control = glmerControl(optimizer = "bobyqa", optCtrl
                           = list(maxfun = 2e6), tolPwrss = 1e-4), nAGQ = 10),

  m_combined_gamlss    = glmer(Disease ~ Combined_Zscores_GAMLSS   + (1 | Study_Location),
                            data = covid, family = binomial, control = glmerControl(optimizer = "bobyqa",
                            optCtrl  = list(maxfun = 2e6), Pwrss = 1e-4), nAGQ = 10),


m_combined_munkholm  = glmer(Disease ~ Combined_Zscores_Munkholm+ (1 | Study_Location),
                      data = covid, family = binomial, control = glmerControl(optimizer = "bobyqa", optCtrl  =
                      list(maxfun = 2e6), tolPwrss = 1e-4), nAGQ = 10)

)
#—— 5) give them readable names for output

cand_models_named <- list(
"TLC Z-scores, GLI equations, (Hall et al. 2021)"                    = cand_models$m_tlc,
  "FEV1/FVC Z-scores, GLI equations, (Quanjer et al. 2012)"         = cand_models$m_ratio,
  "FVC Z-scores, GLI equations, (Quanjer et al. 2012)"               = cand_models$m_fvc,
  "FEV1 Z-scores, GLI equations, (Quanjer et al. 2012)"              = cand_models$m_fev1,
  "VA Z-scores, GLI equations (Stanojevic et al. 2017)"              = cand_models$m_va_gli,
  "VA Z-scores, SLR, (Zavorsky & Cao 2022)"                          = cand_models$m_va_segmented,
  "VA Z-scores, GAMLSS, (Zavorsky & Cao 2022)"                       = cand_models$m_va_gamlss,
  "VA Z-scores, Munkholm et al. (2018)"                              =cand_models$m_va_munkholm,
  "DLCO Z-scores, GLI equations, (Stanojevic et al. 2017)"          = cand_models$m_dlco_gli,
  "DLCO Z-scores, SLR, (Zavorsky & Cao 2022)"                        =
cand_models$m_dlco_segmented,
  "DLCO Z-scores, GAMLSS, (Zavorsky & Cao 2022)"                     = cand_models$m_dlco_gamlss,
  "DLCO Z-scores, Munkholm et al. (2018)"                            = cand_models$m_dlco_munkholm,
  "DLNO Z-scores, SLR, (Zavorsky & Cao 2022)"                        =
cand_models$m_dlno_segmented,
  "DLNO Z-scores, GAMLSS, (Zavorsky & Cao 2022)"                     = cand_models$m_dlno_gamlss,
  "DLNO Z-scores, Munkholm et al. (2018)"                            = cand_models$m_dlno_munkholm,
  "Combined Z-scores, SLR, (Zavorsky & Cao 2022)"                    =
cand_models$m_combined_segmented,
  "Combined Z-scores, GAMLSS, (Zavorsky & Cao 2022)"                 =
cand_models$m_combined_gamlss,
  "Combined Z-scores, Munkholm et al.(2018)"                         =
cand_models$m_combined_munkholm
)
```

```r
#── 6) helper: compute AUROC, 95% CI, Youden threshold
get_auc_metrics <- function(model) {
  prob    <- predict(model, type = "response")
  truth   <- as.integer(covid$Disease)       # now a plain 0/1 integer
  roc_obj <- pROC::roc(truth, prob, quiet = TRUE)

  tibble(
    auroc       = as.numeric(auc(roc_obj)),
    ci_lower    =    ci(roc_obj)[1],
    ci_upper    =    ci(roc_obj)[3],
    best_thresh = as.numeric(
      coords(roc_obj, "best", best.method = "youden")["threshold"]
    )
  )
}
#── 7) compute AUROC results **sequentially**
results_df <- purrr::map_dfr(cand_models_named, get_auc_metrics, .id = "Model")

#── 8) DeLong pairwise tests vs. DLNO-GAMLSS reference
ref_roc <- pROC::roc(
  as.integer(covid$Disease),
  predict(cand_models_named[["DLNO Z-scores, GAMLSS, (Zavorsky & Cao 2022)"]],
          type = "response"),
  quiet = TRUE
)
delong_results <- purrr::map_dfr(
  cand_models_named,
  function(model) {
    test_roc <- pROC::roc(
      as.integer(covid$Disease),
      predict(model, type = "response"),
      quiet = TRUE
    )
    p_val <- tryCatch(
      roc.test(ref_roc, test_roc, method = "delong")$p.value,
      error = function(e) NA_real_
    )
    tibble(p_value = p_val)
  },
  .id = "Model"
) %>%
  mutate(
    p_adj        = p.adjust(p_value, method = "BH"),
    significance = case_when(
      p_adj < 0.001 ~ "***",
      p_adj < 0.01  ~ "**",
      p_adj < 0.05  ~ "*",
      TRUE          ~ ""
    )
  )
```

#—— 9) merge, round, and write to Excel [note: the location where the results are saved is dependent on where the user will save the results]

```
final_output <- left_join(results_df, delong_results, by = "Model") %>%
  arrange(desc(auroc)) %>%
  mutate(across(where(is.numeric), ~ round(.x, 3)))

print(final_output)

output_path <- "C:/Users/gzavorsky/OneDrive - UC Davis Health/Current Working Manuscripts/COVID-19 paper/models_AUROC1.xlsx"

write.xlsx(final_output, file = output_path, asTable = TRUE)
message("
```

```
"DLCO Z-scores, SLR, (Zavorsky & Cao 2022)" = Disease ~ DLCO_Zscores_Segmented + (1 |
 Study_Location),
"DLCO Z-scores, GAMLSS, (Zavorsky & Cao 2022)" = Disease ~ DLCO_Zscores_GAMLSS + (1 |
 Study_Location),
"DLCO Z-scores, Munkholm et al. (2018)" = Disease ~ DLCO_Zscores_Munkholm + (1 |
 Study_Location),
"DLNO Z-scores, SLR, (Zavorsky & Cao 2022)" = Disease ~ DLNO_Zscores_Segmented + (1 |
 Study_Location),
"DLNO Z-scores, GAMLSS, (Zavorsky & Cao 2022)" = Disease ~ DLNO_Zscores_GAMLSS + (1 |
 Study_Location),
“DLNO Z-scores, Munkholm et al. (2018)" = Disease ~ DLNO_Zscores_Munkholm + (1 |
Study_Location),
 "Combined Z-scores, SLR, (Zavorsky & Cao 2022)" = Disease ~ Combined_Zscores_Segmented + (1
| Study_Location),
"Combined Z-scores, GAMLSS, (Zavorsky & Cao 2022)" = Disease ~ Combined_Zscores_GAMLSS +
        (1 |  Study_Location),

   "Combined Z-scores, Munkholm et al.(2018)" = Disease ~ Combined_Zscores_Munkholm + (1 |
        Study_Location)

  )

# 3 Create CV splits
cv_splits <- vfold_cv(covid, v = 5, repeats = 5, strata = Disease)

#4  Compute metrics
compute_metrics <- function(TP, TN, FP, FN) {
  mcc_num <- TP * TN - FP * FN
  mcc_den <- sqrt((TP + FP) * (TP + FN) * (TN + FP) * (TN + FN))
  mcc <- ifelse(mcc_den == 0, NA, mcc_num / mcc_den)

  sensitivity <- ifelse((TP + FN) == 0, NA, TP / (TP + FN))
  specificity <- ifelse((TN + FP) == 0, NA, TN / (TN + FP))
  ppv <- ifelse((TP + FP) == 0, NA, TP / (TP + FP))
  npv <- ifelse((TN + FN) == 0, NA, TN / (TN + FN))
  fdr <- ifelse((TP + FP) == 0, NA, FP / (TP + FP))
  fnr <- ifelse((TP + FN) == 0, NA, FN / (TP + FN))
  forr <- ifelse((FN + TN) == 0, NA, FN / (FN + TN))
  acc <- (TP + TN) / (TP + TN + FP + FN)
  bal_acc <- (sensitivity + specificity) / 2
  tibble(
    MCC = mcc,
    Sensitivity = sensitivity,
    Specificity = specificity,
    PPV = ppv,
    NPV = npv,
    FDR = fdr,
    FNR = fnr,
    FOR = forr,
    Accuracy = acc,
    Balanced_Accuracy = bal_acc
```

```
  )
}

# 5 Evaluate one split
compute_metrics_for_split <- function(split, model_formula, data) {
  train_data <- analysis(split)
  test_data  <- assessment(split)

  model <- tryCatch({
    glmer(model_formula, data = train_data, family = binomial,
          control = glmerControl(optimizer = "bobyqa", optCtrl = list(maxfun = 2e5)))
  }, error = function(e) return(NULL))

  if (is.null(model)) return(NULL)

  train_probs <- predict(model, type = "response")
  train_actual <- train_data$Disease
  roc_obj <- tryCatch({
    pROC::roc(response = train_actual, predictor = train_probs, quiet = TRUE)
  }, error = function(e) return(NULL))

  if (is.null(roc_obj)) return(NULL)

  threshold <- coords(roc_obj, x = "best", best.method = "youden", transpose = TRUE)["threshold"]
  test_probs <- predict(model, newdata = test_data, type = "response", allow.new.levels = TRUE)
  preds <- as.integer(test_probs >= threshold)
  true <- test_data$Disease

  TP <- sum(preds == 1 & true == 1)
  TN <- sum(preds == 0 & true == 0)
  FP <- sum(preds == 1 & true == 0)
  FN <- sum(preds == 0 & true == 1)

  compute_metrics(TP, TN, FP, FN)
}
# 6 Compute per-split metrics for all models
model_metrics <- map_dfr(model_formulas, function(formula) {
  all_metrics <- map(cv_splits$splits, compute_metrics_for_split, model_formula = formula, data =
covid)
  metrics_df <- bind_rows(all_metrics)
  model_name <- names(model_formulas)[[which(model_formulas == formula)]]
  metrics_df$Model <- model_name
  metrics_df
}, .id = NULL)

# 7 Summary stats per model
cv_summary <- model_metrics %>%
  group_by(Model) %>%
  summarise(
    MCC_Mean = mean(MCC, na.rm = TRUE),
    MCC_Lower = quantile(MCC, 0.025, na.rm = TRUE),
```

```
    MCC_Upper = quantile(MCC, 0.975, na.rm = TRUE),
    Sensitivity = mean(Sensitivity, na.rm = TRUE),
    Specificity = mean(Specificity, na.rm = TRUE),
    PPV = mean(PPV, na.rm = TRUE),
    NPV = mean(NPV, na.rm = TRUE),
    FDR = mean(FDR, na.rm = TRUE),
    FNR = mean(FNR, na.rm = TRUE),
    FOR = mean(FOR, na.rm = TRUE),
    Accuracy = mean(Accuracy, na.rm = TRUE),
    Balanced_Accuracy = mean(Balanced_Accuracy, na.rm = TRUE),
    .groups = "drop"
  ) %>%
  arrange(desc(MCC_Mean))

# 8 BH-FDR testing vs GAMLSS reference
ref_model <- "Combined Z-scores, GAMLSS, (Zavorsky & Cao 2022)"
ref_mccs <- model_metrics %>%
  filter(Model == ref_model) %>%
  pull(MCC)

mcc_tests <- model_metrics %>%
  filter(Model != ref_model) %>%
  group_by(Model) %>%
  summarise(
    p_value = tryCatch({
      wilcox.test(MCC, ref_mccs, paired = TRUE)$p.value
    }, error = function(e) NA_real_),
    .groups = "drop"
  ) %>%
  mutate(
    p_adj = p.adjust(p_value, method = "BH"),
    significance = case_when(
      is.na(p_adj) ~ "",
      p_adj < 0.001 ~ "***",
      p_adj < 0.01  ~ "**",
      p_adj < 0.05  ~ "*",
      TRUE ~ ""
    )
  )

# 9 Add reference model manually
ref_row <- tibble(Model = ref_model, p_value = NA_real_, p_adj = NA_real_, significance = "")
mcc_tests <- bind_rows(mcc_tests, ref_row)

# 10 Final merge
cv_summary_fdr <- left_join(cv_summary, mcc_tests, by = "Model") %>%
  arrange(desc(MCC_Mean))

# 11 Output summaries
print(cv_summary_fdr |> select(Model, MCC_Mean, p_value, p_adj, significance), n = Inf)
```

```
# Save to Excel [note: the location where the results are saved is dependent on where the user will save the results]
write.xlsx(cv_summary_fdr, "C:/Users/gzavorsky/OneDrive - UC Davis Health/Current Working Manuscripts/COVID-19 paper/models_MCC_YoudenCV_BH1.xlsx", asTable = TRUE)
message("
```

```
  ) %>%
  arrange(desc(MCC_Mean)) %>%
  mutate(Rank = row_number())

# 5 Rename and process AUROC columns
auroc_df <- auroc_df %>%
  rename(
    AUROC = auroc,
    AUROC_CI_Lower = ci_lower,
    AUROC_CI_Upper = ci_upper,
    AUROC_Threshold = `best_thresh`, # Youden's J
    AUROC_p_value = `p_value`,
    AUROC_p_adj = p_adj,
    AUROC_significance = significance
  )

# 6 Merge both into a single data frame
combined_df <- mcc_df %>%
  select(Model, MCC_Mean, MCC_CI_Lower, MCC_CI_Upper, MCC_p_adj, MCC_significance, Rank)
%>%
  left_join(
    auroc_df %>%
      select(Model, AUROC, AUROC_CI_Lower, AUROC_CI_Upper, AUROC_Threshold,
AUROC_significance),
    by = "Model"
  ) %>%
  mutate(
    CustomLabel = paste0(Rank, ". ", Model),
    Model = factor(Model, levels = rev(unique(Model)))
  )

# 7 Create AUROC + MCC plot
plot <- ggplot(combined_df, aes(x = reorder(CustomLabel, -Rank))) +

  # 8  AUROC bars and error bars
  geom_col(aes(y = AUROC), fill = "steelblue", alpha = 0.7) +
  geom_errorbar(aes(ymin = AUROC_CI_Lower, ymax = AUROC_CI_Upper), width = 0.20, linewidth =
0.8) +

  #9 MCC points and error bars
  geom_point(aes(y = MCC_Mean), color = "darkred", size = 5) +
  geom_errorbar(aes(ymin = MCC_CI_Lower, ymax = MCC_CI_Upper, y = MCC_Mean), color =
"darkred", width = 0.2, linewidth = 0.8) +

  # 10 MCC significance stars (above MCC error bars)
  geom_text(aes(label = MCC_significance, y = MCC_CI_Upper + 0.1), color = "darkred", size = 7,
fontface = "bold", na.rm = TRUE) +

  #11 AUROC significance stars (above AUROC error bars)
  geom_text(aes(label = AUROC_significance, y = AUROC_CI_Upper + 0.1), color = "black", size = 7,
fontface = "bold", na.rm = TRUE) +
```

```
  coord_flip() +
  geom_hline(yintercept = 0, color = "black", linewidth = 0.8) +
  labs(title = "AUROC and MCC by Model (Ranked by MCC)", x = "Model (MCC by Rank)", y = "AUROC
/ MCC") +
  scale_y_continuous(
    limits = c(0, 1),
    breaks = seq(0.0, 1, by = 0.125),
    sec.axis = sec_axis(~., name = "AUROC / MCC", breaks = seq(0, 1, by = 0.125))
  ) +
  theme_minimal(base_size = 18) +
  theme(
    axis.text.y = element_text(size = 20, hjust = 0),
    axis.text.x = element_text(size = 20),          # Bottom x-axis
    axis.text.x.top = element_text(size = 20),       # Top x-axis

    axis.title = element_text(size = 20),
    plot.title = element_text(size = 20, hjust = 0.5),
    axis.ticks.length = unit(0.3, "cm"),
    axis.ticks.y = element_blank(),
    panel.grid.major.y = element_blank(),
    panel.grid.minor.y = element_blank(),
    axis.ticks.x.bottom = element_line(color = "black", linewidth = 0.8),
    axis.ticks.x.top = element_line(color = "black", linewidth = 0.8),
    axis.line.x.top = element_line(color = "black", linewidth = 0.8),
    axis.line.x.bottom = element_line(color = "black", linewidth = 0.8),
    plot.background = element_rect(fill = "white", color = NA),
    panel.background = element_rect(fill = "white", color = NA)
  )
#12 Save the plot
file_path <- "C:/Users/gzavorsky/OneDrive - UC Davis Health/Current Working Manuscripts/COVID-19
paper/Figures/FigureMCC_AUROC.tiff"
ggsave(
  file_path,
  plot = plot,
  width = 19,
  height = 12,
  dpi = 600,
  compression = "lzw",
  device = "tiff"
)
# 13 Save merged data [note: the location where the excel file will be saved is dependent on
where the user will save the results]
combined_path <- "C:/Users/gzavorsky/OneDrive - UC Davis Health/Current Working
Manuscripts/COVID-19 paper/models_MCC_AUROC_combined1.xlsx"
openxlsx::write.xlsx(combined_df, file = combined_path, asTable = TRUE)

# 14 Console output
message("
```

Polyserial correlations between the modified Medical Research Council dyspnea score (mMRC, 0 to 4), and pulmonary function test z-scores.

```
# ------------------------------------------------------------------------------------------------------------------------------------
# Polyserial correlations ranked by most negative to least negative
# ------------------------------------------------------------------------------------------------------------------------------------
#Load the file:  Dyspnea_vs_diffusing_capacity

# 1 polyserial correlations ranked my most negative to least negative
# Load necessary libraries
library(polycor)  # For polyserial correlations
library(dplyr)    # For data manipulation
library(future)   # For parallel processing
library(furrr)    # For parallel mapping

# 2 Set up parallel processing with 14 cores and set a seed for reproducibility
plan(multisession, workers = 14)
set.seed(123)  # Set seed for reproducible parallel processing

# 3 Filter data to remove rows where MRC_Dyspnea is NA
Covid <- Dyspnea_vs_diffusing_capacity %>%
  filter(!is.na(MRC_Dyspnea))

# 4 List of continuous z-score variables
zscore_vars <- c("Combined_Zscores", "DLCO_Zscores", "DLNO_Zscores", "VA_Zscores",
                 "FEV1_FVC_Ratio_Zscores", "FEV1_Zscores", "FVC_Zscores")

# 5 Map labels for the variables
label_mapping <- c(
  "Combined_Zscores" = "Combined DLNO+DLCO z-scores",
  "DLCO_Zscores" = "DLCO z-scores",
  "DLNO_Zscores" = "DLNO z-scores",
  "VA_Zscores" = "VA z-scores",
  "FEV1_FVC_Ratio_Zscores" = "FEV₁/FVC ratio z-scores",
  "FEV1_Zscores" = "FEV₁ z-scores",
  "FVC_Zscores" = "FVC z-scores"
)

#6  Ensure MRC_Dyspnea is treated as an ordered factor
Covid$MRC_Dyspnea <- as.ordered(Covid$MRC_Dyspnea)

# 7 Compute polyserial correlations with bootstrapping in parallel
results <- future_map(zscore_vars, function(var) {
  if (!var %in% colnames(Covid)) {
    warning(paste("Variable", var, "not found in the dataset."))
    return(NULL)
  }
```

```
# 8 Filter complete cases for the pair
  complete_data <- Covid %>%
    filter(!is.na(MRC_Dyspnea), !is.na(.data[[var]]))

  # 9 Number of non-missing observations
  n_cases <- nrow(complete_data)

  if (n_cases > 2 && length(unique(complete_data$MRC_Dyspnea)) > 1 &&
is.numeric(complete_data[[var]])) {
    # Polyserial correlation
    correlation <- polyserial(complete_data[[var]], complete_data$MRC_Dyspnea)

  # 10 Bootstrap for CI (10,000 samples)
    boot_correlations <- replicate(10000, {
      sample_data <- complete_data[sample(1:n_cases, replace = TRUE), ]
      polyserial(sample_data[[var]], sample_data$MRC_Dyspnea)
    })
    ci_lower <- quantile(boot_correlations, 0.025, na.rm = TRUE)
    ci_upper <- quantile(boot_correlations, 0.975, na.rm = TRUE)

    #11 p-value using t-test
    t_value <- correlation * sqrt((n_cases - 2) / (1 - correlation^2))
    p_value <- 2 * (1 - pt(abs(t_value), df = n_cases - 2))

    # 12 Return results as a data frame
    data.frame(
      Label = label_mapping[var],
      Polyserial_Correlation = round(correlation, 3),
      CI_Lower = round(ci_lower, 3),
      CI_Upper = round(ci_upper, 3),
      P_Value = round(p_value, 3),
      N_Cases = n_cases
    )
  } else {
    warning(paste("Insufficient data or invalid type for", var))
    data.frame(
      Label = label_mapping[var],
      Polyserial_Correlation = NA,
      CI_Lower = NA,
      CI_Upper = NA,
      P_Value = NA,
      N_Cases = n_cases
    )
  }
}, .options = furrr_options(seed = TRUE))

# 13 Combine results into a data frame, excluding NULL entries
results_df <- do.call(rbind, results[!sapply(results, is.null)])

# 14 Sort by Polyserial_Correlation from most negative to least negative
results_df <- results_df[order(results_df$Polyserial_Correlation), ]
```

```
# 15 Remove row names to avoid duplication
row.names(results_df) <- NULL

# Print the sorted results
print(results_df)

#16 Reset parallel processing plan to default
plan(sequential)
# -------------------------------------------------------------------------------------------------------------------------------
# Polyserial correlations Figure
# -------------------------------------------------------------------------------------------------------------------------------
library(ggplot2)
library(dplyr)  # For mutate()

# Add a significance column, reorder labels, and format p-values
results_df <- results_df %>%
  mutate(
    Significance = ifelse(P_Value < 0.05, "Significant", "Non-Significant"),
    Label = factor(Label, levels = Label[order(Polyserial_Correlation)]),
    P_Label = case_when(
      is.na(P_Value) ~ NA_character_,
      P_Value < 0.001 ~ "p < 0.001",  # Explicitly set to plain text "p < 0.0001"
      TRUE ~ sprintf("p = %.3f", P_Value)  # Use plain text for other p-values, no italic
    )
  )
# Create the updated plot
plot1 <- ggplot(results_df, aes(x = Label, y = Polyserial_Correlation, color = Significance)) +
  geom_point(size = 10) +  # Larger circles
  geom_errorbar(aes(ymin = CI_Lower, ymax = CI_Upper), width = 0.4, size = 1, color = "blue") +  #
Error bars
  geom_hline(yintercept = 0, linetype = "dashed", size = 1.5, color = "purple") +  # Horizontal line at 0
  coord_flip() +  # Flip coordinates
  scale_color_manual(values = c("Significant" = "red", "Non-Significant" = "black")) +  # Color scheme
  labs(
    x = "Variables",
    y = "Polyserial Correlations",
    color = "Significance"
  ) +
  scale_y_continuous(breaks = seq(-0.50, 0.10, 0.10)) +  # Adjust y-axis breaks
  geom_text(aes(label = P_Label),
            parse = FALSE,  # Disable parsing to ensure plain text rendering
            vjust = -1.5,  # Raise p-value labels above the error bars
            hjust = 0.5,  # Center the labels horizontally
            size = 8,  # Increase font size of p-values
            color = "black") +  # Text color for p-values
  theme_minimal(base_size = 20) +
  theme(
    panel.background = element_rect(fill = "white", color = NA),
    plot.background = element_rect(fill = "white", color = NA),
    axis.text.x = element_text(size = 20),
```

```
    axis.text.y = element_text(size = 20),
    axis.title.x = element_text(size = 22, face = "bold"),
    axis.title.y = element_text(size = 22, face = "bold"),
    plot.title = element_text(size = 20, face = "bold", hjust = 0.5),
    axis.line.x = element_line(size = 1, color = "black"),
    axis.line.y = element_line(size = 1, color = "black"),
    axis.ticks.length = unit(0.3, "cm"),
    axis.ticks.x = element_line(size = 0.8),
    axis.ticks.y = element_line(size = 0.8),
    legend.text = element_text(size = 20),  # Increase legend font size
    legend.title = element_text(size = 22),  # Increase legend title font size
    legend.position = "none", # remove legend
  )
# Print the plot
print(plot1)

# Save the updated plot [note: the location where the figure will be saved is dependent on where the user will save the results]

file_path <- "C:/Users/gzavorsky/OneDrive - UC Davis Health/Current Working Manuscripts/COVID-19 paper/Figures/polyserial.tiff"

ggsave(file_path, plot = plot1, width = 15, height = 12, dpi = 600, compression = "lzw", device = "tiff")

# ------------------------------------------------------------------------------------------------------------------
# Boxplot Figure: Combined DLNO+DLCO z-scores and dyspnea scores (ordinal, from 0 to 4)
# ------------------------------------------------------------------------------------------------------------------

#1 Prepare data with numeric dyspnea score
Covid <- Dyspnea_vs_diffusing_capacity %>%
  filter(!is.na(MRC_Dyspnea)) %>%
  mutate(
    MRC_Dyspnea = factor(MRC_Dyspnea, levels = 0:4),
    Dyspnea_Numeric = as.numeric(as.character(MRC_Dyspnea))
  )
# Wilcoxon pairwise stats
pairwise_stats <- Covid %>%
  wilcox_test(Combined_Zscores ~ MRC_Dyspnea, p.adjust.method = "BH")

# Add y-position and asterisks
y_positions <- seq(15, 55, length.out = nrow(pairwise_stats))

pairwise_stats <- pairwise_stats %>%
  mutate(
    group1 = as.numeric(as.character(group1)),
    group2 = as.numeric(as.character(group2)),
    y.position = y_positions,
```

```
  Asterisk_Label = case_when(
   p.adj < 0.001 ~ "***",
   p.adj < 0.01 ~ "**",
   p.adj < 0.05 ~ "*",
   TRUE ~ "ns"
  ),
  label_y = y.position + 2,   # 👈 Shift labels 2 units above brackets
  blank_label = ""            # 👈 Used to suppress fallback labels
 )

# Median z-scores by dyspnea level
medians <- Covid %>%
 group_by(Dyspnea_Numeric) %>%
 summarize(Median_Z = median(Combined_Zscores, na.rm = TRUE), .groups = "drop")

# Plot
plot2 <- ggplot(Covid, aes(x = Dyspnea_Numeric, y = Combined_Zscores)) +
 geom_boxplot(
  aes(group = Dyspnea_Numeric),
  outlier.shape = NA, fill = "white", color = "black", size = 1.5
 ) +
 stat_summary(
  fun = median, geom = "segment",
  aes(xend = Dyspnea_Numeric, yend = ..y.., group = Dyspnea_Numeric),
  fun.data = median_hilow,
  fun.args = list(conf.int = 0),
  size = 0.6, color = "black"
 ) +
 geom_jitter(
  width = 0.2,
  size = 4.5,
  alpha = 0.35,
  color = "black"     # Darkest possible grey
 ) +
 geom_line(
  data = medians,
  aes(x = Dyspnea_Numeric, y = Median_Z),
  color = "red", size = 2
 ) +
 geom_point(
  data = medians,
  aes(x = Dyspnea_Numeric, y = Median_Z),
  shape = 21,         # Circle with border
  fill = "red",       # Inside color
```

```
  color = "black",       # Border color
  size = 6,
  stroke = 1.2          # Border thickness
) +
geom_text(
  data = pairwise_stats,
  aes(x = (group1 + group2) / 2, y = label_y, label = Asterisk_Label),
  size = 12,
  color = "blue",
  fontface = "bold"
) +
stat_pvalue_manual(
  pairwise_stats,
  label = "blank_label",  # 👈 No visible label here
  tip.length = 0.01,
  bracket.size = 1.3,
  hide.ns = FALSE,
  color = "blue",
  parse = FALSE
) +
scale_x_continuous(
  breaks = 0:4,
  labels = as.character(0:4),
  name = "Dyspnea score"
) +
scale_y_continuous(
  limits = c(-11, 57),  # 👈 increased y-limit slightly for extra spacing
  breaks = seq(-10, 55, 5)
) +
labs(y = "Summed DLNO+DLCO z-scores") +
theme_minimal(base_size = 20) +
theme(
  panel.background = element_rect(fill = "white", color = NA),
  plot.background = element_rect(fill = "white", color = NA),
  axis.text.x = element_text(size = 22),
  axis.text.y = element_text(size = 22),
  axis.title.x = element_text(size = 22, face = "bold"),
  axis.title.y = element_text(size = 22, face = "bold"),
  axis.line.x = element_line(size = 1, color = "black"),
  axis.line.y = element_line(size = 1, color = "black"),
  axis.ticks.length = unit(0.3, "cm"),
  axis.ticks.x = element_line(size = 0.8),
  axis.ticks.y = element_line(size = 0.8),
  legend.text = element_text(size = 20),
```

```
  legend.title = element_text(size = 22)
 )
# Show plot
print(plot2)

# Save to TIFF [note: the location where the figure will be saved is dependent on where the user will save the results]
ggsave(
 filename = "C:/Users/gzavorsky/OneDrive - UC Davis Health/Current Working Manuscripts/COVID-19 paper/Figures/Boxplot.tiff",
 plot = plot2,
 width = 15,
 height = 12,
 units = "in",
 dpi = 600,
 compression = "lzw",
 device = "tiff"
)
```

#Combine plot 1 and plot 2 in one figure (side by side)

```
#1 Load libraries
library(ggplot2)
library(patchwork)

# Make sure both plot1 and plot2 are defined earlier in your script

# Example (REPLACE with your actual plot1 and plot2 code):
# plot1 <- ggplot(data1, aes(x, y)) + geom_point() + theme_minimal()
# plot2 <- ggplot(data2, aes(x, y)) + geom_boxplot() + theme_minimal()

# Combine side-by-side
combined_plot <- plot1 + plot2 + plot_layout(ncol = 2)

# Save combined plot [note: the location where the figure will be saved is dependent on where the user will save the results]

ggsave(
  filename = "C:/Users/gzavorsky/OneDrive - UC Davis Health/Current Working Manuscripts/COVID-19 paper/Figures/Polyserial_and_Boxplot.tiff",
  plot = combined_plot,
  width = 18,          # total width of both plots
  height = 12,         # consistent height
  units = "in",
  dpi = 600,
  compression = "lzw",  device = "tiff")
```

```
#------------------------------------------------------------------------------------------------------------------------
#Spearman's correlations
#------------------------------------------------------------------------------------------------------------------------

#1 Load necessary libraries
library(dplyr)

# 2 Assume Covid, zscore_vars, and label_mapping are defined from previous code; If not,
redefine them here:

Covid <- Dyspnea_vs_diffusing_capacity %>% filter(!is.na(MRC_Dyspnea))
zscore_vars <- c("Combined_Zscores", "DLCO_Zscores", "DLNO_Zscores", "VA_Zscores",
                 "FEV1_FVC_Ratio_Zscores", "FEV1_Zscores", "FVC_Zscores")
label_mapping <- c(
  "Combined_Zscores" = "Combined DLNO+DLCO z-scores",
  "DLCO_Zscores" = "DLCO z-scores",
  "DLNO_Zscores" = "DLNO z-scores",
  "VA_Zscores" = "VA z-scores",
  "FEV1_FVC_Ratio_Zscores" = "FEV₁/FVC ratio z-scores",
  "FEV1_Zscores" = "FEV₁ z-scores",
  "FVC_Zscores" = "FVC z-scores"
)

# 3 Compute Spearman correlations for each z-score variable
spearman_results <- lapply(zscore_vars, function(var) {
  # Check if the variable exists in the dataset
  if (!var %in% colnames(Covid)) {
    warning(paste("Variable", var, "not found in the dataset."))
    return(NULL)
  }

  # 4 Filter complete cases for the pair
  complete_data <- Covid %>%
    filter(!is.na(MRC_Dyspnea), !is.na(.data[[var]]))

  # 5 Check if there's enough data
  if (nrow(complete_data) < 2) {
    warning(paste("Insufficient data for", var, "to compute Spearman correlation."))
    return(NULL)
  }

  # 6 Ensure z-score variable is numeric
  if (!is.numeric(complete_data[[var]])) {
    warning(paste("Variable", var, "is not numeric."))
    return(NULL)
  }

  # 7 Convert MRC_Dyspnea to numeric for Spearman correlation
  dysp_numeric <- as.numeric(complete_data$MRC_Dyspnea)
```

```
# 8 Compute Spearman correlation
  correlation <- cor(complete_data[[var]], dysp_numeric, method = "spearman", use = "complete.obs")

# 9 Return a data frame with results
  data.frame(
    Label = label_mapping[var],
    Spearman_Correlation = correlation
  )
})

# 10 Combine results into a data frame, excluding NULL entries
spearman_results_df <- do.call(rbind, spearman_results[!sapply(spearman_results, is.null)])

# 11 Round correlations to three decimal places
spearman_results_df$Spearman_Correlation <- round(spearman_results_df$Spearman_Correlation, 3)

# 12 Sort by Spearman_Correlation from most negative to least negative
spearman_results_df <- spearman_results_df[order(spearman_results_df$Spearman_Correlation), ]

# 13 Remove row names for clean output
row.names(spearman_results_df) <- NULL

# 14 Display the sorted Spearman correlations
print(spearman_results_df)
```

Bayesian mixed-effects proportional odds logistic regression analysis to model the relationship between various lung function z-scores and the ordinal outcome MRC_Dyspnea

#Load the file: Dyspnea_vs_diffusing_capacity

Then,

```
# 1. Load Libraries
library(brms)
library(dplyr)
library(posterior)
library(tibble)
library(purrr)

#2 Set up parallel processing with 14 cores and set a seed for reproducibility
plan(multisession, workers = 14)
set.seed(123)  # Set seed for reproducible parallel processing

#3  Filter data to remove rows where MRC_Dyspnea is NA
Covid <- Dyspnea_vs_diffusing_capacity %>%
  filter(!is.na(MRC_Dyspnea))

# 4. Setup: variable names, factor structure
zscore_vars <- c("Combined_Zscores", "DLCO_Zscores", "DLNO_Zscores", "VA_Zscores",
                 "FEV1_FVC_Ratio_Zscores", "FEV1_Zscores", "FVC_Zscores")

Covid$MRC_Dyspnea <- as.ordered(Covid$MRC_Dyspnea)

if (!"Study" %in% names(Covid)) {
  stop("Column 'Study' not found in dataset.")
}
# 5. Fit models
ordinal_models <- lapply(zscore_vars, function(var) {
  form <- bf(reformulate(c(var, "(1 | Study)"), response = "MRC_Dyspnea"))
  brm(
    formula = form,
    data = Covid,
    family = cumulative("logit"),
    chains = 4, iter = 5000, warmup = 2000,
    cores = 14, seed = 123,
    refresh = 0, silent = TRUE
  )})
names(ordinal_models) <- zscore_vars
```

```
# 6. Summarization Function
summarize_model <- function(model, var_name) {
  if (!inherits(model, "brmsfit")) return(NULL)

  fix <- fixef(model)
  row <- fix[var_name, , drop = FALSE]

  or <- exp(row[, "Estimate"])
  or_ci <- exp(row[, c("Q2.5", "Q97.5")])
  re_var <- VarCorr(model)$Study$sd[1, "Estimate"]^2

  draws <- as_draws_df(model)
  re_sd <- draws[[grep("^sd_Study__Intercept", names(draws), value = TRUE)]]

  if (is.null(re_sd)) {
    warning(paste("Random effect SD not found for", var_name))
    return(NULL)
  }

  #7 Compute ICC manually
  re_var_post <- re_sd^2
  icc_post <- re_var_post / (re_var_post + (pi^2 / 3))
  icc_summary <- quantile(icc_post, probs = c(0.025, 0.5, 0.975), na.rm = TRUE)

  tibble(
    Variable = var_name,
    Coefficient_Estimate = round(row[, "Estimate"], 4),
    Std_Error = round(row[, "Est.Error"], 4),
    CI_Lower_LogOdds = round(row[, "Q2.5"], 4),
    CI_Upper_LogOdds = round(row[, "Q97.5"], 4),
    Odds_Ratio = round(or, 4),
    OR_CI_Lower = round(or_ci[1], 4),
    OR_CI_Upper = round(or_ci[2], 4),
    Random_Effect_Variance = round(re_var, 4),
    ICC_Median = round(icc_summary[2], 4),
    ICC_Lower = round(icc_summary[1], 4),
    ICC_Upper = round(icc_summary[3], 4)
  )
}

# 8. Apply summarization
model_summaries <- map2(ordinal_models, zscore_vars, summarize_model)
results_df <- bind_rows(model_summaries)

# 9. Print results
cat("\nBayesian Mixed-Effects Proportional Odds Model Results\n")
print(results_df, row.names = FALSE)
```

References

1. [dataset] Zavorsky GS, Barisioni G, Gille T, et al. "The long term effects of COVID-19 on pulmonary diffusing capacity for nitric oxide (DLNO) and carbon monoxide (DLCO)", Mendeley Data, V3, doi: 10.17632/92gvt9vmrm.3, 2025.
2. Barisione G, Brusasco V. Lung diffusing capacity for nitric oxide and carbon monoxide following mild-to-severe COVID-19. *Physiol Rep* 2021;9(4):e14748. doi: 10.14814/phy2.14748 [published Online First: 2021/02/25]
3. Barisione G, Brusasco V. Lung diffusing capacities for nitric oxide and carbon monoxide at rest and post-walking in long COVID. *ERJ Open Res* 2023;9(2) doi: 10.1183/23120541.00363-2022 [published Online First: 20230417]
4. Seccombe LM, Heath D, Farah CS, et al. Mechanisms of gas transfer impairment utilizing nitric oxide following severe COVID-19 pneumonitis. *Physiol Rep* 2023;11(7):e15660. doi: 10.14814/phy2.15660
5. Imeri G, Conti C, Caroli A, et al. Gas exchange abnormalities in Long COVID are driven by the alteration of the vascular component. *Multidiscip Respir Med* 2024;19(1) doi: 10.5826/mrm.2024.938 [published Online First: 20240306]
6. Nunez-Fernandez M, Ramos-Hernandez C, Garcia-Rio F, et al. Alterations in Respiratory Function Test Three Months after Hospitalisation for COVID-19 Pneumonia: Value of Determining Nitric Oxide Diffusion. *J Clin Med* 2021;10(10) doi: 10.3390/jcm10102119 [published Online First: 2021/06/03]
7. Dal Negro RW, Turco P, Povero M. Long-lasting dyspnoea in patients otherwise clinically and radiologically recovered from COVID pneumonia: a probe for checking persisting disorders in capillary lung volume as a cause. *Multidiscip Respir Med* 2022;17:875. doi: https://doi.org/10.4081/mrm.2022.875
8. Lytzen AA, Helt TW, Christensen J, et al. Pulmonary diffusing capacity for carbon monoxide and nitric oxide after COVID-19: A prospective cohort study (the SECURe study). *Exp Physiol* 2024 doi: 10.1113/EP091757 [published Online First: 20240326]
9. Agostoni P, Mapelli M, Salvioni E, et al. Symptomatic post COVID patients have impaired alveolar capillary membrane function and high VE/VCO_2. *Respir Res* 2024;25(1):82. doi: 10.1186/s12931-023-02602-3 [published Online First: 20240208]
10. Sesé L, Beurnier A, Schlemmer F, et al. Pathophysiological insights from lung diffusing capacity for nitric monoxide (DLNO) after COVID-19. *European Respiratory Journal* 2022;60(suppl 66):2662. doi: 10.1183/13993003.congress-2022.2662
11. Sesé L, Uzunhan Y, Khamis W, et al. Contribution of the DLCO-DLNO double diffusion technique in the evaluation of post-COVID respiratory functional sequelae [FRENCH]. *Revue des Maladies Respiratoires Actualités* 2022;14(1):138–39. doi: https://doi.org/10.1016/j.rmra.2021.11.209
